# A Gestalt Theory Approach to Structure in Language

**DOI:** 10.3389/fpsyg.2021.649384

**Published:** 2021-06-18

**Authors:** Hans Buffart, Haike Jacobs

**Affiliations:** ^1^Retired, Krefeld, Germany; ^2^Centre for Language Studies, Radboud University, Nijmegen, Netherlands

**Keywords:** human cognition, the magical number, universal grammar, usage-based grammar, syntactic embeddedness, phonological structure, consonant shift, dissimilation

## Abstract

The fact that human language is highly structured and that, moreover, the way it is structured shows striking similarities in the world’s languages has been addressed from two different perspectives. The first, and more traditional, generative hypothesis is that the similarities are due to an innate language faculty. There is an inborn ‘grammar’ with universal principles that manifest themselves in each language and cross-linguistic variation arises due to a different parameter setting of universal principles. A second perspective is that there is no inborn, innate language faculty, but that instead structure emerges from language usage. This paper purports to develop and illustrate a third perspective, according to which the structural similarities in human languages are the result of the way the cognitive system works in perception. The essential claim is that structural properties follow from the limitations of human cognition in focus.

## Introduction

The paper is structured as follows. In section “Structure in Language,” we will concentrate on the fact that human language is highly structured. This section will be more useful for readers who have no linguistic background than for those readers who are familiar with linguistics. In section “Toward a Gestalt-like approach to structure in language,” we will review the essential elements of this third perspective. This model fits within the so-called Gestalt psychology and emphasizes that it is the observer who creates the experienced structures when, as a subject, she operates on the environment. The cognitive system is described as a set of operations on its environment. A subset of this set is Focus which approaches the elements in the environment in a sequential way and governs the visual and aural experience. According to the model, Focus capacity is restricted to a maximum of seven elements, a restriction that forces the structuring of perceived elements. After that, section “Applications” presents applications of the model on different linguistic both syntactic and phonological phenomena. We argue that the limits of Focus capacity rather than the limitations of memory account for the fact that center-embedding is more complicated than initial or final embedding and we provide a different answer to the question why lenition interactions are typically counter-feeding. Section “Discussion,” finally, presents a conclusion and some discussion and indicates possible directions for future research.

## Structure in Language

### Dual Structure in Language

One of the most striking aspects of all human languages (estimates are about 7000) is that language is highly structured. Every language is characterized by what the French linguist André Martinet has termed ‘l’articulation double’ or dual structure. That is, every utterance has both a morphosyntactic structure, conveying what the expression means, as well as phonological structure, how the utterance is pronounced. The first type of structure is most clearly seen in cases of structural or syntactic ambiguity, like the sentence in (1).


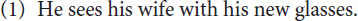


**FIGURE 1:**
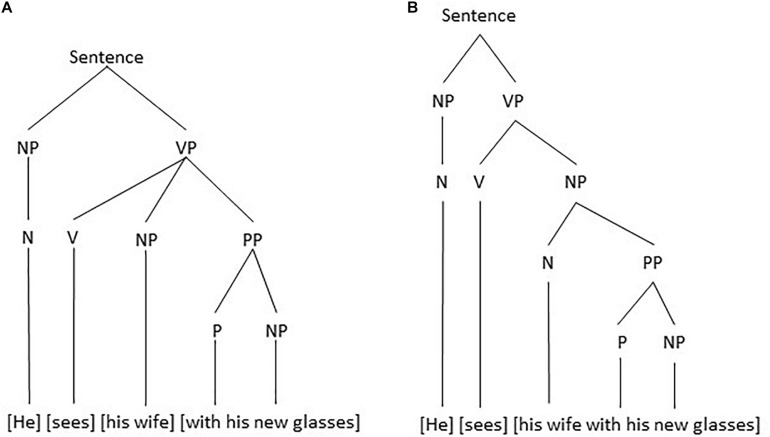
Two possible morphosyntactic structures: **(A)** the verb has one complement, which can be moved to the initial position, and **(B)** the verb has two complements, each of which can be fronted.

The double meaning is not due to the individual meaning of the words in the sentence, but due to a different structuring of the sentence. Either there is a subject who is seeing and an object (his wife) as well as an instrument with which the subject sees (his new glasses) or there is a subject who is seeing and an object (his wife carrying/trying out etc. his new glasses). The two possible morphosyntactic structures might be visualized in the form of tree diagrams as the ones in Figure 1.

In Figure 1 NP stands for noun phrase, i.e., a group of words of which the noun is the obligatory part (like *he* and *his wife*), a VP stands for a verb phrase with obligatorily a verb (like *sees*), and a PP for a preposition phrase with a preposition (like *with* in *with his glasses*). The particular structure in Figure 1 is motivated by the assumption that only one element or group can be moved to the initial position. In Figure 1A the verb has one complement which can be moved producing sentence (2a), which has the same meaning. In Figure 1B the verb has two complements each of which can be fronted, but not the two of them together. So, for Figure 1B the sentences in (2b-c) are acceptable, but not the one in (2a) which has a different meaning, that is, no longer two complements (an object and an instrument), but one single complement.


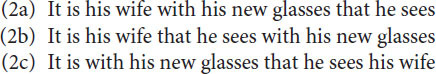


The second structure, the phonological structure, is most clearly seen in cases where it is not isomorphic with the morphosyntactic structure. Consider for instance the French sentence in (3).


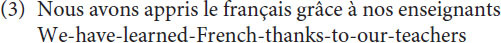


**FIGURE 2:**
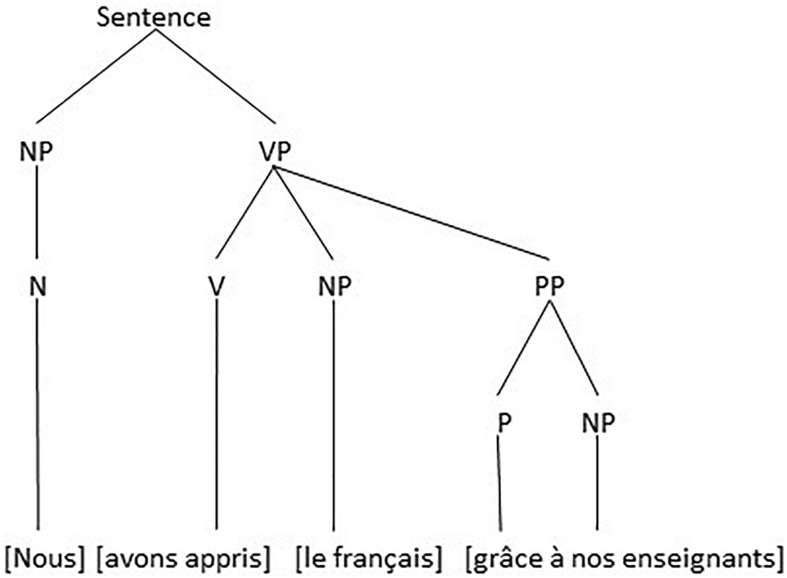
The Morphosyntactic structure of utterance (3).

The morphosyntactic structure of utterance (3) is clear. There are an NP (*nous*) a VP (*avons appris le français grâce à nos enseignants*) which contains a Verb (*avons appris*) an NP (*le français*) and a PP (*grâce à nos enseignants*), visualized as in Figure 2.

**FIGURE 3:**
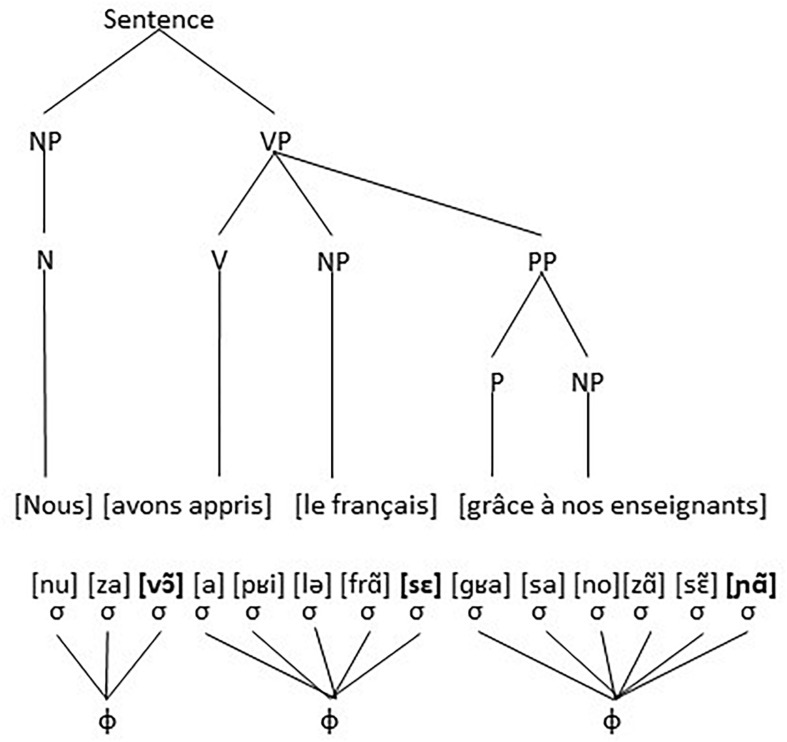
The grammatical structure (above) does not coincide with the phonological structure (below).

However, the phonological structure of utterance (3), reflecting its pronunciation, is grouped differently. There are 14 syllables [nu] [za] [vɔ͂] [a] [pʁi] [lə] [frã] [sε] [ɡʁa] [sa] [no] [zã] [sɛ͂] [nã]. These 14 syllables are further grouped into three (or possibly two depending on speech rate) accentual groups or phonological phrases, where the final syllable (and possible the initial syllable) of each accentual group is characterized by being more prominent (stronger and stressed) than the preceding syllables, which is indicated by boldface. The three possible groups are: ([nu] [za] **[vɔ͂]**), ([a] [pʁi] [lə] [frã] **[sε]**), and ([ɡʁa] [sa] [no][zã] [sɛ͂] **[nã]**). There is clearly no one-to-one mapping between units of the morphosyntactic structure and units of the phonological structure. The noun *Nous* or the preposition *grâce* for instance are one element, one morpheme, an N and a P, in the morphosyntactic structure, but divide over two syllables in the phonological structure. Similarly, the verb *avons appris* is one single unit, an inflected verb, in the morphosyntactic structure, but is divided over two accentual groups or phonological phrases in the phonological structure. The utterance with the two structures is visualized in Figure 3, where the Greek letter σ stands for syllable and the Greek letter φ for phonological phrase.

In this section, we have seen that human language is structured and that languages have a dual structure. The fundamental question is, where does this structure come from, in the next section we will further investigate the two types of linguistic structure.

### Structure in Language and the Magical Number 7-2

Miller (1956) reported on perception experiments involving tone height and observed that seven is an upper limit when it comes to discriminating and classifying different tones. This is consistent with a general use in psychology of seven-point rating scales, given the intuition that adding more or trying to rate into finer categories does not add much to the usefulness of the ratings. With respect to the tones experiments he remarks: “[the] results [up to six different classes HB/HJ] indicate that, at least for pitches, this intuition is fairly sound. […] if you can discriminate five high-pitched tones in one series and five low-pitched tones in another series, it is reasonable to expect that you could combine all ten into a single series and still tell them all apart without error. When you try it, however, it does not work. The channel capacity for pitch seems to be about six and that is the best you can do.” The number seven is also mentioned by Martin (2011), who illustrated its relevance for the second type of structure discussed above, the phonological structure. If a sound string in French^1^ is longer than seven syllables, the listener is forced to group the string in higher-order constituents, that form accentual groups. An accentual group, therefore, is never larger than seven syllables. Also, if an utterance exceeds seven accentual groups, a break is required, both in oral and in written language. In (4) this is illustrated with an example taken from Martin (2011).

The utterance in (4a) consists of 32 syllables, as indicated in (4b) where the dots mark syllable boundaries. Given that an accentual group has an upper limit of seven syllables, there need to be, minimally five accentual groups. The way the utterance was actually realized shows the relevance of the number 5. It was divided into eight accentual groups, with each group containing at most five syllables, indicated by the square brackets with the number of syllables the group contains, as illustrated in (4b). Notice that since the actual way in which the utterance was realized has more than seven accentual groups, a pause is required, which was indeed realized after the 6th accentual group.


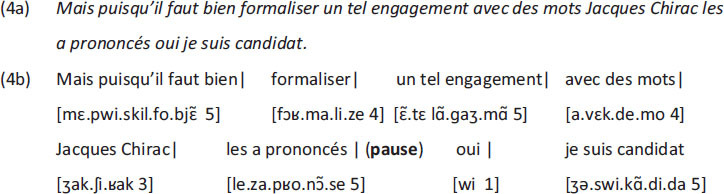


The division of individual sounds into syllables and the psycholinguistic reality of syllables is motivated by the existence of language games where, for instance, the syllables within a word are inverted, as in French Verlan, so that *parents* ‘parents’ [pa.rã] or *vacances* [va.kãs], for instance, are realized as *rentspa* [rã.pa] and *cancesva* [kãz.va]. If speakers could not gain implicit awareness of the way they divide words into syllables, they would not be able to manipulate them in a language game. Slips of the tongue, like Spooner’s “queer old dean” instead of “dear old queen,” in a similar way, show that the segmentation of the speech signal, that is decoding the acoustic signal into a series of discrete sounds and in syllables, is a psycho-linguistic reality. If speakers did not decode the acoustic signal, in which, in fact, there is continuous overlap between sounds and in which neither syllables nor segments are physically present as discrete elements or units, they would not be able to invert single sounds.

The discrete sounds that listeners perceive are not atomic elements but are themselves further internally structured. One of the most important discoveries of structuralism linguistics in the 20th century is without any doubt the phoneme. Sounds differ in whether they are meaningful elements or not. If they are meaningful, they are called phonemes, if not, they are considered contextual variants of a sound and are called allophones. In French, the sounds [l] and [r] can appear in the same phonological contexts, finally, as in *le bal* ‘the ball’ and *le bar* ‘the bar,’ initially, as in *lire* ‘to read’ and *rire* ‘to laugh,’ and intervocalically, as in *le boulot* ‘the work’ and *le bourreau* ‘the hangman.’ The difference between the two sounds is the only difference in the pronunciation of each pair of words. The distribution of the two sounds is free, in the sense that the occurrence of the one or the other is not determined by the position in the word. In a language like Korean, the distribution of the two sounds is not free, but contextually determined. In intervocalic position only [r] occurs, but never [l]. In other positions (initially or finally) only [l] occurs, but never [r]. In Korean, the occurrence of [r] is thus determined by the position in the word. As such, the two sounds in Korean can never form meaningful distinctions, whereas they can do that in French. The two sounds are thus phonemes in French but are allophones, that is, contextually determined variants of a single phoneme, in Korean. The phoneme was considered not to be an atomic unit, but rather was argued to consist of a set of distinctive features, such as [±voice] referring to presence or absence of vocal fold vibration or [±continuant], referring to continuous airflow or complete obstruction of it.

The next two examples show the psycholinguistic relevance of distinctive features. At a normal speech rate, the first consonant of the Dutch verbs ‘to go,’ ‘to find’ and ‘to sit’ are pronounced as [ɣ], [v] and [z] in (5a), but as [x], [f], and [s] in (5b). On the other hand, the last consonant of the 1st person singular pronoun *ik* is pronounced, not as [k] as in (5b), but as [g] in (5c).


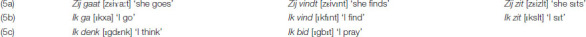


In (5b) we observe progressive voice assimilation, the second consonant, that is the [ɣ], [v] and [z], assimilates to the first consonant, the [k], and takes over its characteristic of being unvoiced. In (5c), there is regressive voice assimilation, the first consonant, the [k], assimilates to the voicing of the second one, the [d] and the [b], and takes over its characteristic of being voiced. The sounds represented by the letters *g*, *z* and *v*, that is, [ɣ], [z] and [v], have two features in common, [+voice] referring to vocal fold vibration and [+continuant] referring to continuous airflow. In Dutch the combination of these two features is shared by these three sounds, but by no other Dutch sound, which is what specifies them as a group. As a group they are treated in a systematically different way from other sounds by Dutch speakers. Similarly, the two sounds that are involved in triggering regressive assimilation in (5c), the [b] and the [d], share the features [+voice] and [–continuant] which again sets them off as a group against all other consonants of Dutch. Importantly, if sounds were treated by speakers as atomic units, there would be no way in which the grouping of the sounds [d], [b] and the sounds [ɣ], [z], and [v], into precisely these two groups, the voiced plosives and the voiced fricatives would be a natural or a logical one.

**FIGURE 4:**
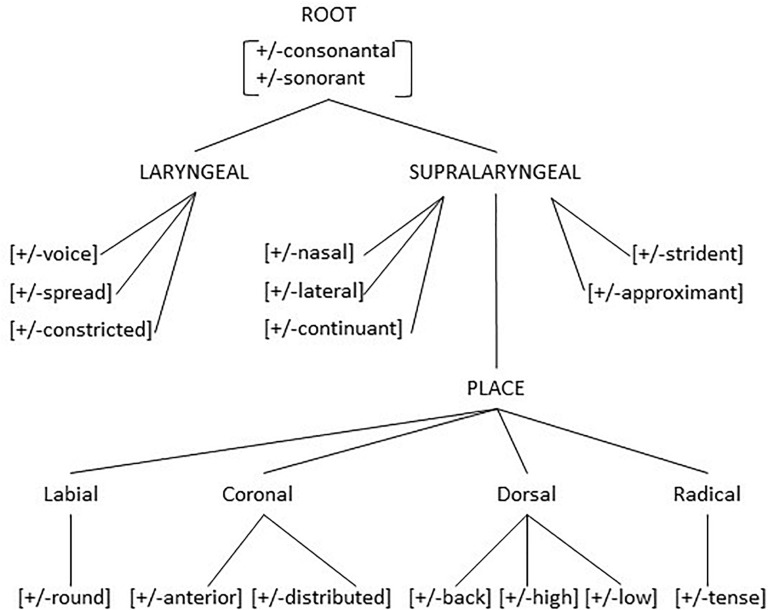
Feature geometry.

The distinctive features themselves are grouped into subsets which can be visualized as in Figure 4 (Gussenhoven and Jacobs, 2017, p. 238). The motivation for this grouping of the distinctive features into subsets, again is motivated on the basis of possible modifications that groups of sounds undergo, as in the examples in (5). Such modifications in languages may target a single feature or a single subset of features, but never refer to two subsets at the same time. For features the number 7 shows also up. For the coronal sounds, the two features allow for four contrastive sounds^2^ and for six possible contrasts. Clements (2009) observes that a phonetic theory which recognizes the phonetic categories, that is the possible places of articulation (dental and alveolar) and the possible tongue shapes (apical/tip or laminal/blade), ‘apicodental,’ ‘apicoalveolar,’ ‘laminodental,’ ‘laminoalveolar,’ ‘palatoalveolar,’ ‘retroflex,’ and ‘palatal’ allows for 7 sounds and 21 possible contrasts. Up to this point, we have primarily focused on spoken language. But it should be noticed that the same duality of structure and a similar internal structure along the lines of Figure 4 above also exist in sign language. We refer to, among others, Sandler (1993); van der Hulst (1993), and Crasborn and van der Kooij (2013) for some further discussion of the internal phonological representation of signs.

So to summarize, the fact that language is structured is evident from the morphosyntactic sentence structure, as in Figure 1, from the division of strings into accentual groups, from the division of accentual groups into syllables, from the division of syllables into discrete categories of vowels and consonant and from the division of individual sounds into groups of features. Besides recurring structure there is also the number 5 that is recurring. It showed up in the grouping of sentence (4a) into eight accentual groups of five syllables each, and, given the eight accentual groups, larger than seven, the necessity of a pause after six groups. Upper limit 7 and the number 5 show up in the five main categories of sentence structure (NP, VP, AP, PP, AdvP), but also in the upper limit of consonants in a syllable. Georgian (Butskhrikidze, 2002) has unusually long sequences of consonants, such as /prckvn^3^ /, /mc’vrtn/, /brt’X’/ in words like /prckvna/ ‘to peel,’ /mc’vrtneli/ ‘trainer,’ /brt’X’eli/ ‘flat.’ The longest sequence of consonants in the initial position of a syllable is maximally 5.

The role of the number 5 shows also up in the internal structure of the individual segment. If five determines an upper limit of structuring, the features given in Figure 4, a total number of 21 need to be further structured. And they indeed are as indicated in Figure 4. Evidence for the internal structure again can be provided by the way human speakers systematically modify the sounds of their languages when pronouncing them in a given context, as exemplified in (5) above. It should also be mentioned that not all the features in Figure 4 are present in every language. The model in Figure 4 allows to characterize any sound of any human language and any group of sounds that in a language behaves in systematically the same way as a natural class, that is, as a collection of sounds identifiable as a single subset of Figure 4. For instance, in the example in (5) above what is modified is the LARYNGEAL node of the consonants involved.

### Awareness of Structures in Language

Human beings have no explicit knowledge of the fact that language is highly structured nor of the psychological processes that are involved when they speak. As Roberts (2017) so eloquently puts it: “We are mostly as blissfully unaware of the intricacies of the structure of language as fish are of the water they swim in. We live in a mental ocean of nouns, verbs, quantifiers, morphemes, vowels and other rich, strange and deeply fascinating linguistic objects.”

Although speakers are unaware of, for instance, the dual structure of language, the mere fact that speakers of English are able to grasp the two meanings of the sentence in (1) above, shows that they have awareness of the first type of structure. Moreover, native speakers have clear intuitions about what is or what is not acceptable or grammatical in terms of words and sentences in their language. Dutch native speakers will all agree that a sentence like *Morgen komt Piet* ‘tomorrow-comes-Piet’ is acceptable, but a sentence like *Morgen Piet komt* ‘tomorrow-Piet-comes’ is not. These sentences, if translated into French, will be judged differently by native French speakers. They will all agree that a sentence like *Demain vient Pierre* is unacceptable, whereas *Demain Pierre vient* is not. Similarly, as mentioned above, the ability to invert the syllables of a word (in language games) or parts of the syllable (in slips of the tongue) shows that perceiving the acoustic signal as discrete sounds and in syllables is a cognitive process. If speakers did not decode the acoustic signal, in which there is continuous overlap between sounds and in which neither syllables nor segments are physically present as discrete elements or units, they would not be able to invert single sounds. Moreover, whereas speech production is highly variable and gradient, speech perception is categorical and not gradient. For instance, the Spanish minimal pair *gasa* ‘gas’ and *casa* ‘house’ in a concrete recording of a female native speaker of Spanish shows in the acoustic signal a difference of a negative voice onset time for the [g] of –80 ms. This means that the vocal folds start to vibrate 1/10 of a second before the release of the closure. For the [k] a 10 ms positive VOT was measured, meaning that the vocal folds start to vibrate 1/100 of a second after the release of the closure. Taking steps and reducing the negative VOT by 20 ms per step leads to a switching point at around –20, where respondents start hearing it as [k]. At –60 and at –40 they still perceive it as [g]. Importantly, they will always perceive it as either [g] or [k], but never as something in between. Where does this categorical perception come from, and where does the structure in general and most importantly the upper limit of 5–7 come from? We will address that question in the next paragraph.

### Where Does Structure Come From?

The main goal of theoretical linguistics since the 60’s of the previous century has been to characterize the tacit linguistic knowledge, the linguistic competence of native speakers. (1965: 200) observes that “It seems clear that many children acquire first or second languages quite successfully even though no special care is taken to teach them and no special attention is given to their progress. It also seems apparent that much of the actual speech observed consists of fragments and deviant expressions of a variety of sorts. Thus it seems that a child must have the ability to “invent” a generative grammar that defines well-formedness and assigns interpretations to sentences even though the primary linguistic data that he uses as a basis for this act of theory construction may, from the point of view of the theory he constructs, be deficient in various respects.” Chomsky’s explanation for the rapidity with which children are able to acquire any human language that they are exposed to thus is that humans have an inborn, innate language faculty or language acquisition device for which the input is the language exposed to and for which the output is the grammar or the competence of the language being acquired. This innate language faculty, or language instinct (Pinker, 1994), is traditionally termed Universal Grammar (UG). Focusing on distinctive features, the assumption of an innate set of distinctive features (around 20 all in all) is advocated by for instance Hale and Reiss (2008). Over the last decade the assumption of an innate language faculty has been subject to a lot of debate (cf. for instance, among others, Evans and Levinson, 2009; Levinson and Evans, 2010). The main reason for doubting an innate language faculty or an innate set of distinctive features was based on the observation that structural aspects claimed to be universal were not attested in every language. To give just two examples, recursion, the backbone of UG (Hauser et al., 2002) has been claimed not to exist in Pirahã (cf. discussions in Everett, 2005; Nevins et al., 2009) and the syllable has been argued to be absent as a relevant notion in Gokana (Hyman, 2011) or Japanese (Labrune, 2012).

The interesting question that then arises is where does structure come from if not from UG? Where does phonological knowledge, such as the categorization of the acoustic signal into a string of discrete segments containing distinctive features, come from? If we dismiss the Chomskyan UG hypothesis and the universal set of distinctive features it contains (in the Hale and Reiss, 2008 view) the structures (syntactic categories, morphosyntactic structure, syllable structure and the internal structure of sounds) that we observe on the basis of linguistic evidence have to come from somewhere else. The typical answer provided by those who reject the innatist view, is that it comes from, or emerges from, language usage rather than being part of UG (Johnson, 1997; Bybee, 2006; van de Weijer, 2014; Van de Weijer, 2017; Archangeli and Pulleyblank, 2015; and many others). Although there are a lot of different usage-based approaches to language, such as, among others, Construction Grammar (cf. for instance Tomasello, 2003; Goldberg, 2006) and Exemplar-Theory (cf., among others, Johnson, 1997; Pierrehumbert, 2001), they all share the assumption that there is no innate universal grammar, but that instead, grammatical knowledge emerges in usage. It would be far beyond the scope of the present paper to give a comprehensive overview of the different approaches. For a more detailed discussion of the two different approaches (innate vs. usage-based) we refer to Dąbrowska (2015) and Pleyer and Hartmann (2019), who discuss, with respect to language evolution, to what extent the two approaches show divergences as well as convergences. Here, we will just limit ourselves to one particular illustration (van de Weijer, 2014, which is based on Exemplar theory). The key idea of Exemplar theory is that individual manifestations of aspects of experience are stored and leave true traces in memory. New instances are compared against stored traces. “Every token of experience is classified and placed in a vast organizational network as part of the decoding process. New tokens of experience are not decoded and discarded, but rather they impact memory representations. In particular, a token of linguistic experience that is identical to an existing exemplar is mapped onto that exemplar, strengthening it. Tokens that are similar but not identical (differing in slight ways in meaning, phonetic shape, pragmatics) to existing exemplars are represented as exemplars themselves and are stored near similar exemplars to constitute clusters or categories.” (Bybee, 2006, p. 716).

van de Weijer (2014) provides the following example. In Dutch, a certain group of consonants, that is [v], [z], [b], [d], and [ɣ], is realized, in final position, as [f], [s], [p], [t], and [x]. The first person singular and plural of verbs like *wij geven* and *ik geef* ‘we give, I give’ *wij lezen* and *ik lees* ‘we read, I read,’ *wij hebben* and *ik heb* ‘we have, I have,’ *wij wedden* and *ik wed* ‘we bet, I bet’ and *wij zeggen* and *ik zeg* ‘we say, I say’ differ precisely in this respect, that is a voiceless consonant in the singular verb forms, but a voiced one in the plural forms. Words that in the plural form do not have a voiced consonant, like *boffen* ‘to be lucky,’ *wensen* ‘to wish,’ *meppen* ‘to bang,’ *zetten* ‘to put down’ or *lachen* ‘to laugh’ have the same consonant in the singular and plural forms.

From an innatist point of view, there is an innate feature [voice] which is part of the innate universal phonological vocabulary and a language-specific rule of final devoicing, or for that matter, two universal constraints, both part of UG. A constraint IDENTITY [VOICE], requiring that input and output forms are identical with respect to the feature value for voice, that is [+voice] should surface as [+voice] and [–voice] as [–voice] and a constraint which only allows voiceless obstruents in final position, ^∗^VOICED-CODA. Depending on the relative ranking of these two constraints, two language types can be identified. If ^∗^VOICED-CODA is more important than IDENTITY [VOICE] a language with final devoicing results, if not one without. For Dutch the former ranking applies, for English the latter. The Dutch language learner only needs to memorize one consonant, that is the one that occurs in the plural form, and can derive or compute the alternating one by the phonological grammar.

**FIGURE 5:**
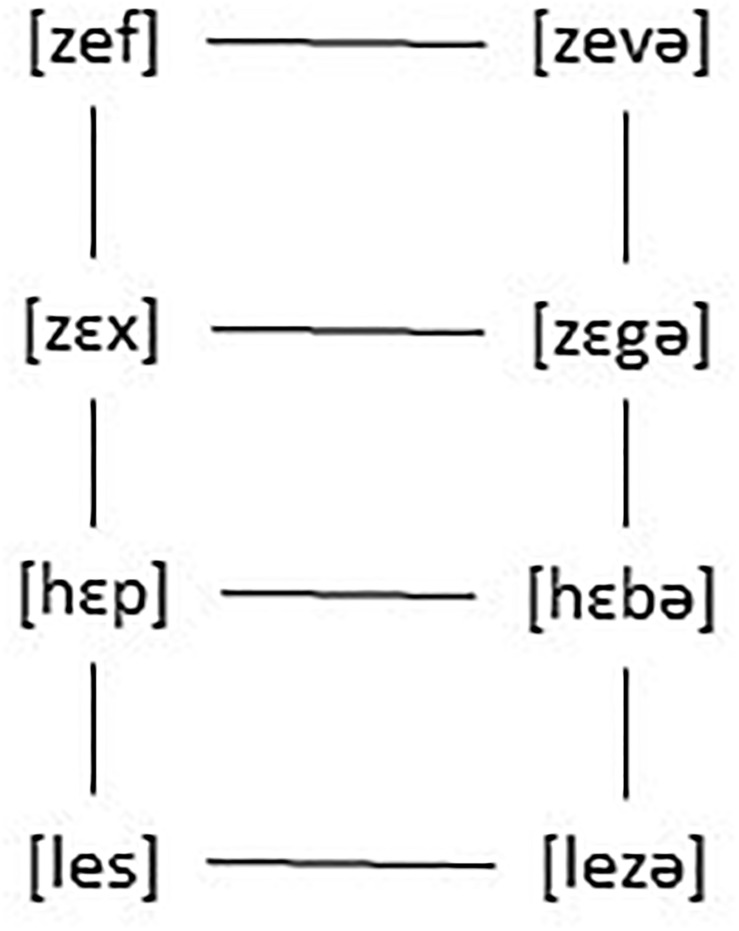
A cloud of tokens and relations between forms.

In an ET (Exemplar Theory) account it works as follows. Every heard form is stored which leads to forms as a cloud of tokens and to relations between forms such as the ones in Figure 5.

The stored alternations will lead speakers to discover the generalization that in final position no voiced obstruents are allowed. Constraints such as IDENTITY [VOICE] and ^∗^VOICED-CODA can thus, as argued for by van de Weijer (2014) be derived from exposure to the language or emerge in language use, but are not innate, being learnable on the basis of date, they emerge naturally. But why would that be required, if everything is stored? Why is there structure at all in language? Why are sign languages structured and why do they not use only iconic signs? Why does spoken language not consist of unstructured vocalizations coupled with morphemes (Gussenhoven and Jacobs, 2017, p. 11)? Why is there categorical perception if slightly different ways in phonetic shape are stored?

In the next section, we will develop our own view, which takes sides with usage-based approaches in the sense that exposure to language is required for the learning of language-specific structure to arise (such, as for instance whether it is VO or OV), but differs to the extent that structure as such is not derived from exposure to language, but is a direct result of the way in which the cognitive system works in perception. Bybee (2010) argues that the structure emerges from abstractions of language use by domain general cognitive processes such as “chunking, rich memory storage, analogy and cross-modal associations” (Bybee, 2010, p. 7). Chunking is the process triggering the formation and use of formulaic or prefabricated sequences of words, such as for instance *eat your heart out*, and is the basic mechanism responsible for the formation of constructions and constituent structure. The cognitive focus model that we will address in the next section not only provides a straightforward answer to the question why chunking exists in language, but also, why language does not consist of unstructured vocalizations coupled with morphemes (Gussenhoven and Jacobs, 2017, p. 11), which in principle could be the case and could be learnable. We will argue that the only way in which cognition can interact with the environment is by imposing structure on it, which is a direct result from the cognitive focus model in section “Toward a Gestalt-like approach to structure in language.” That is, the only way in which cognition can interact with the environment is by imposing structure on it. Christiansen and Chater (2008) define a number of constraints that shape language for each generation of language learners and users and that determine general learning and processing. Besides constraints from thought, perceptuo-motor constraints and pragmatic constraints, they define cognitive constraints on sequential learning and processing. The cognitive focus model that is the topic of the next section can be considered as a more formal account of such cognitive constraints such as chunking.

## Toward a Gestalt-Like Approach to Structure in Language

One may wonder why we discussed in section “Structure in language” such divergent topics which at first sight have nothing to do with each other. However, our claim is that we can explain these phenomena based on one and the same theory. Buffart (2017) formulated its principles. We will discuss his starting points and his conclusions without going into the details of his proofs since these are of a purely mathematical nature.

### General Principles

There are two different views on the way humans process the outside world. One view basically considers the human system as a sort of information processing mechanism. More specifically, the system receives stimulation or input and processes this. In this way, the structures in the outside world are projected into the inside. Research in this area has emphasized the mechanism of information processing. It is outside-in. The other, opposing, view is basically one that is inside-out. It emphasizes that humans build structures. Research in this area has emphasized the experience of structure.

Naturally, both views have a lot in common. The outside-in view does not deny that humans interpret the environment and the second view does not deny that there are structures outside and that there is some type of processing in the brain. However, the theoretical presuppositions completely differ. The brain processing model of Buffart (2017), which will be used in this paper, has been based on the second view. It fits within the so-called Gestalt psychology which finds its roots in the publications of Mach (1886) and von Ehrenfels (1890). Gestalt theory emphasizes that it is the observer who creates the experienced structures (Koffka, 1935; Köhler, 1947, 1969). Thus, a subject operates on its environment.

**FIGURE 6:**
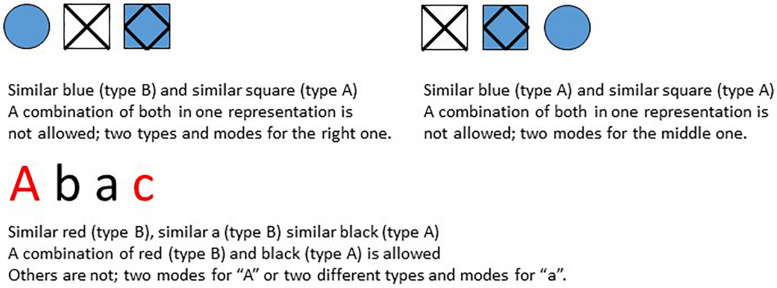
Types of modes and relations.

Buffart (2017) assumes that a subject focuses when operating on its surroundings^4^. He further assumes that a subject/system in focus^5^ experiences a sequence of elements in its surroundings. The surroundings and thus the elements are not specified. Elements are sets of operations on its surroundings as well, for example a set that refers to the experience of a tree, a lion, a letter or a square. Within a sequence a subject can experience relations. A relation between two elements does not mean that these elements are similar. It only means that under some viewpoint these elements have something in common. Examples can be seen in Figure 6.

A subject builds representations. A representation is a set (the whole) of relations between the elements (the parts) of a sequence. There are different modes of relations, such as “blue,” “square,” “red” and “a” in Figure 6. For a mode there are two types of position relations. Elements can be neighbors, let us call it type A relations, or non-neighbors, let us call it type B relations (see Figure 6). If two non-neighbors have a relation, all elements in between are treated as one element at the current surface level. For example, p, f and l in apfla are grouped together a(pfl)a.

A basic assumption is that in a Focus-representation there is no interference between different modes and different types, which means that in a representation an element can be involved in only one type and only one mode. So, let in the following sequences G represent elements that are in some way (e.g., same color, content or sound) related to each other. Then the sequence GGHG can be described by two types of representation relations: type A, *GG* followed by two elements (HG) and type B, *G*(GH)*G* with two concatenated elements in between. In a more abstract way: If we denote the related elements by X and the non-related elements by Y and Z, valid representations are *XX*YZ (type A; ***GG***HG), *X*(YZ)*X* (type B; ***G***(GH)***G***), and Y*XZX* (type B as well; G***G***H***G***), where X, Y, and Z refer to elements G or H depending on their position. Notice that in this case the type A representation together with a type B representation covers the structure of the whole sequence XXZX, as do both type B representations together as well. For example, XXYZ means the first and second elements are related and X(YZ)X means the first and last elements are related, so that both together imply that the first, second and last elements are related. One calls such a pair complementary representations since they cover the structure of the whole sequence.

From these assumptions a mathematical theorem, which we will not explain here (see Buffart, 2017 for a detailed account), follows according to which in every sequence that does not contain more than seven elements all similarities of one mode can be represented with at most two complementary representations. For sequences that have more than seven elements one needs in some cases more than two representations. Moreover, representations with 5, 6, or 7 elements have similar characteristics. Representations with fewer elements differ. Buffart observed that this finding is remarkable since 5 up to 7, or 7 minus 2, as discussed in section “Structure in language and the magical number 7–2” above, is an often discussed question in cognition and language research and the occurrence of two representations at the same time is known in visual perception research as duality (Rock, 1977) or complementarity (van Tuijl and Leeuwenberg, 1979). On the basis of these facts, Buffart states that in Focus each representation has a maximum length of seven and maximal two representations can be active together. This statement only holds for Focus.

### Focus Versus Memory

Buffart (2017) defined the cognitive system as a set of operations on its surroundings. This surroundings can be the world outside the body but also the memory content, being operations as well. Focus has been introduced as a subset of this set, of which the operations approach the elements in the surroundings in a sequential way. It governs the “inside-outside” experience. The role of Focus is comparable with the role of “Apperception” as Wundt (1903) describes it. Due to the mathematical results described in the foregoing section Focus capacity has been restricted to a maximum of seven elements. This restriction forces structure, called representation. Since even the elements in a representation are “inside” operations, i.e., representations of other experiences, Focus also governs the structure of notions, ideas, images, melodies and so on.

In addition to Focus there is Memory. In Memory these representations are stored. Storing means retaining the relations between the elements within a representation and between representations. Since a sequence in Focus can have more than one representation, these representations become connected through their elements. These elements themselves are clusters of representations as well. In this way a network is built. Notice that in Memory several representations can be activated, but in Focus at most two, one of which is the one that is momentarily preferred.

In short, in Focus at most two representations are active and their sequences have maximally seven elements. Systems with a maximum of 5, 6, or 7 elements are indistinguishable with respect to the type of representations. The differences between these systems are processing capacity and number of connections in Memory. In Memory several representations can be active, but in Focus only two, one of which is the most active one. The elements in a representation are (clusters of) representations as well. If a sequence is longer than 5, 6, or 7, Focus will cluster two or more elements into one element. It builds a representation of this shrunken sequence and as a part of it a representation of the cluster. So, not sounds, consonants, letters, words, sentences, melodies or objects are stored in Memory, but their various representations, of which the limitations are dictated by Focus. Focus is the gate between Memory and the “outside” world. It plays its role in perception and production. This corresponds with the basic assumption of Gestalt Psychology that the system is always active, or in short, perception is production.

## Applications

Successful applications of the theoretical principles in vision are known since 1969 (Leeuwenberg, 1969, 1971). We will apply them onto several phenomena in language. We will show that the notion of an innate language faculty is not peculiar, since its core properties, recursion and the merger of elements into higher elements (the language faculty in its narrow sense, Hauser et al., 2002), follow from the limitations of Focus, or to put it differently, from the way the brain works in general. We will show how the Focus properties straightforwardly account for some basic notions of a Universal Grammar. We will give a new explanation of Latin liquid dissimilation. We will develop a way of modeling consonants and so explain the historical and actual shifts between them.

Before discussing these applications in more detail, we would like to emphasize that the theory does not describe any processing other than preference. Focus is a general principle filter that forces the cognitive structures, and processing of these structures takes place in Memory determining what the actual representation is. We will come back to that in the discussion.

### Language

According to the theory, systems with a Focus-capacity from five up to seven possess the same type of operations; they may differ in processing speed and the connectivity in Memory. If communication mechanisms in a society must be usable for all users/humans, their structures must be based on the lowest capacity, a Focus-capacity of five. So, the prediction is, that in almost all spoken or sign-languages the maximum sequence length in terms of elements is five. Given that the theory is essentially based on sequential analyses of the surroundings, it follows that in principle language use is linear, as Frank et al. (2012) stated. We think that all structural aspects of language can be understood as a consequence of Focus-capacity.

After a short remark on the language faculty we will discuss embeddedness since it reveals the difference between Universal Grammars and Focus-theory. Finally, we will argue that the hierarchical structure of sentences is not necessarily stored in Memory.

#### The Language Faculty

Due to the Focus limitation language expressions are broken up in general into maximal five elements, which could themselves be expressions as well. Since the Focus limitations are an inborn property, scientists, following the line of thought as laid down in Chomsky (1965), who state that natural languages are structured and that this rests on an innate property, are right. It is an inborn property in the sense that the restriction of Focus is an inborn property. Its limitations enforce structure. Parsing a sentence, for example, shows a division in five parts: subject, verb, direct object, indirect object and adjuncts. The assumption of an inborn language faculty, however, is not warranted. Moreover, structure does not necessarily imply a generative grammar. Generative grammar in principle implies that center-embedding is as natural as final embedding, also called cross-dependency recursion, and that the depth of embedding is unlimited. Karlsson (2007, 2010) and Christiansen and Chater (2015) have shown on the basis of a corpus of written speech that there is a limit to center-embedding. The logical depth might be unlimited (Chomsky and Miller, 1963), but the cognitive depth isn’t. Karlsson (2010) states that the overall results on multiple center-embedding mainly depends on the limitations of working memory. We, instead, would say on Focus-capacity.

#### Views on Embeddedness

The classical textbook example of center-embedding is the sentence in (6a) (Chomsky and Miller, 1963, p. 286). Its final embedded counterpart is the sentence in (6b). A sentence like (6a) may seem harder to process than a sentence like (6b), but within generative grammar there are no grammatical restrictions against (6a).


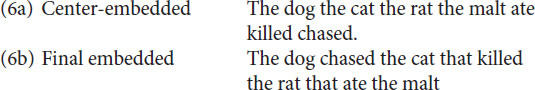


We will discuss the problem of embeddedness on the basis of some Dutch examples. Dutch has been traditionally analyzed (Koster, 1975) as being underlying an SOV language. In Dutch, the V is the second element of a main clause, but follows the complements in a subordinate clause, as illustrated in (7).


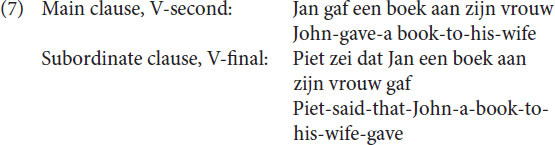


This means that both sentences in (7) in that particular analysis in generative grammar are base generated as having an OV structure, the surface structure of main clauses is then derived by placing the V of the main clause in second position. This implies that a final embedded sentence like (8a) is base generated as in (8b).


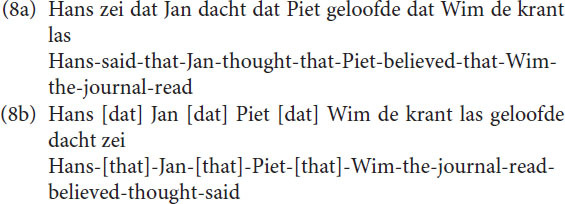


**FIGURE 7:**
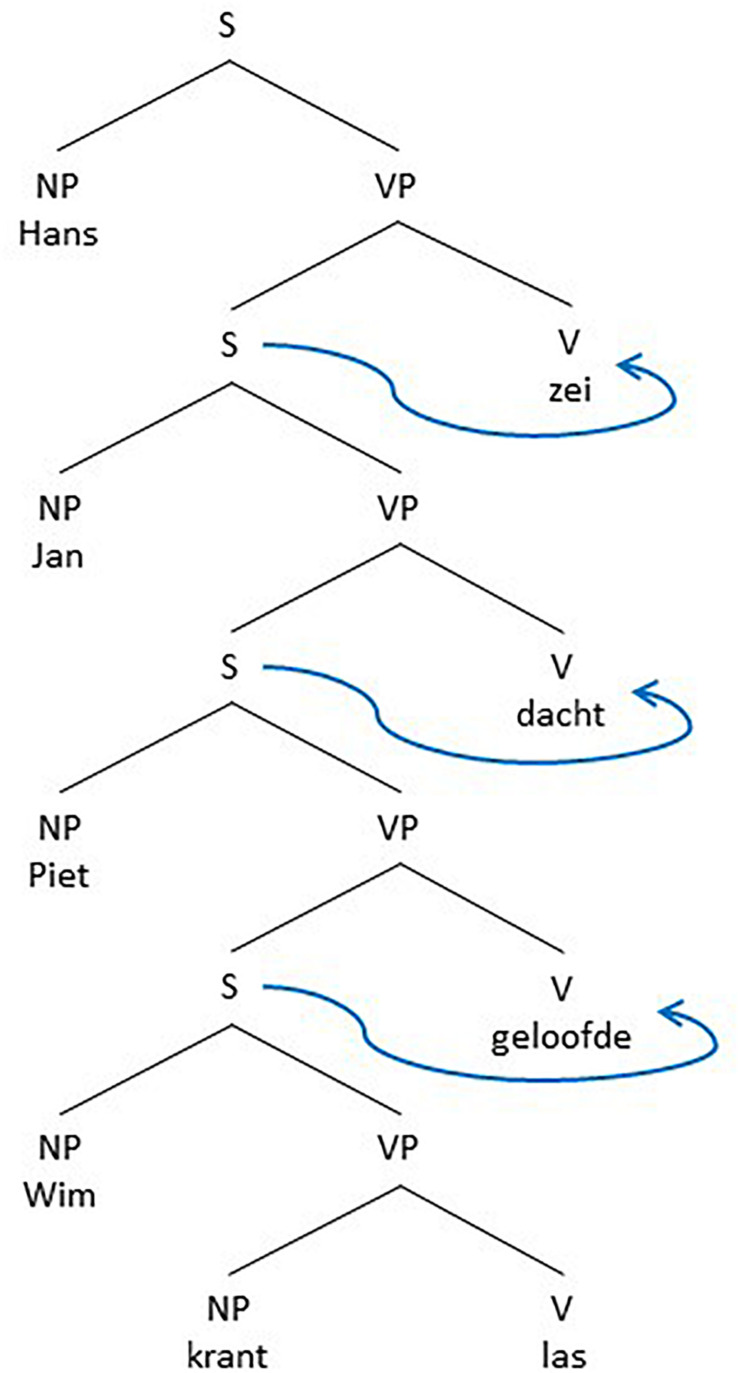
A reduced structure of utterance (8b) and its transformation into utterance (8a).

The underlying reduced and simplified structure is given in Figure 7. In order to get the actual final embedded sentence as it is realized in speech, every S in Figure 7, except the first one, needs to be moved (by a transformational rule termed S-Extraposition) with all structure it contains below to the right of the V next to it, as indicated by the blue arrows.

As remarked above, from a generative perspective, generating embedded structures by rewriting a VP as consisting of either a V and an S structure or an S and a V structure, or, as more recently by merging a V and an S or an S and a V in a higher-order VP structure is computationally speaking of the same complexity and therefore predicted to be equally likely. Although this might be true from a speaker or production-based perspective, the situation is quite different from a listener or perception-based perspective. Focus capacity, as we will argue, at the same time both restricts the possibilities of embeddedness and forces the recursion structures in final embeddedness. Focus-capacity also restricts the recursion depth. To illustrate this, we show below seven Dutch examples of recursion, based on variations on the final embedded sentence in (9).


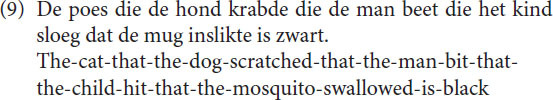


In the examples below, FC means the Focus-capacity needed for parsing the surface structure of a sentence at once. An element of the surface structure is a word or a sub sentence or even a complex structure that a listener must recall to understand the sentence. We use the symbol ≥ to indicate the Focus capacity needed. Given that we do not know the details that a subject really includes, we can only count the minimum capacity needed. Notice the clear difference between the center-embedded recursion in the sentences (9a), (9c), and (9e) and the final embedding, or cross-dependency recursion, in the sentences (9b), (9d), and (9f) respectively.


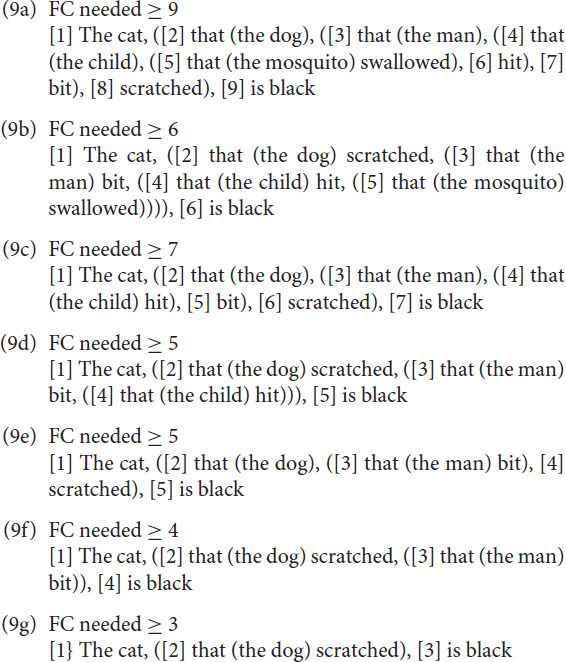


The sentences (9a-c) are problematic. This does not mean that sentences with a recursion depth of 3 or more (4 or more sentences) are impossible. But one can only understand or produce them with the involvement of Memory, evading the restrictions of Focus. As a consequence, one may find them in written language easier than in spoken language. But even in written language the depths are finite (Karlsson, 2007, 2010). The reason for the restricted depth is that Focus operates sequentially. In a similar way as in (9), one can calculate for utterances (8a) and (8b) the minimal FC needed as shown in (10a) and (10b). Again, there is a clear difference between the center-embedded and the final embedded sentence.


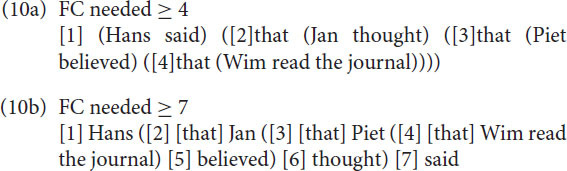


#### The Structure of Language According to Focus

A strong point of the hierarchical description of languages is, that the grouping of elements in one larger element is not arbitrary. Focus has two grouping mechanisms. (1) When neighbors are related (Type A) these elements will form one new element. (2) When two non-neighbor elements have a relation (type B), all elements in between are grouped into one expression. In other words, the building of a new element by combining elements can be understood as a property of Focus. Articles, adjectives, and nouns are strong examples of the first type of grouping mechanism and will be grouped together in one larger element. Recursion is a strong example of the latter kind of grouping. Although the grouping may seem clear for these examples, it is not so clear for situations where the meaning or the context plays a role. Examples are the sentences below. Sentences (11a) and (11b) are ambiguous, which means that there are (at least) two almost equally strong interpretations. The grouping depends on the meaning. If “better” is meant to be related to “with his new glasses” sentence (11c) is a better formulation than (11b), since it is not ambiguous. As a reply to a question like “John wears his new glasses; who does he see?” the grouping in (11a) and (11b) is unambiguous.


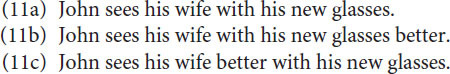


Focus does not prescribe order. From the viewpoint of Focus the sentence “his John glasses new wife his sees with” is as good as the sentence “John sees his wife with his new glasses.” However, Focus has a maximum capacity. It means that the first sentence cannot be used for good communication since grouping is impossible so that the sentence is too long. As shown in Figure 1 one can recognize one or two subgroups in sentence (11a), so that it consists of three or four elements at surface level. As argued, the maximum is five elements; if not, sentences are cognitively inconceivable. This fully determines the grouping and the ordering mechanisms in languages. The most effective way of grouping is placing side by side what belongs to each other, such as grouping preposition article adjectives and the noun, or the words belonging to a subordinate clause, or verb and subject, and verb and direct or indirect object. These groupings guarantee that a surface level contains maximal five elements. Sentence parsing reflects this in that it generally distinguishes five categories (subject, verb, direct object, indirect object, and adjunct). Given these groupings the order is free in principle and one can use propositions to show the role of a noun as in “John gives a book to Mary,” or a language may use order to show this role as in “John gives Mary a book about fishing.” Languages with declension have more freedom of order. Focus handles sentences with a maximum length between 5 and 7 elements. Thus, utterances that do not group what belongs to each other don’t pass Focus. In this sense, Focus forces the preference for order that allows grouping to limit the length of sentences, which as such provides a principled and formal explanation for the claim defended in Futrell et al. (2015) that dependency length minimization is a general property of human information processing. Focus does not specify which order. So, a specific order is not forced by Focus, but is an evolved property of a language and thus usage based. It means that order preferences may differ between languages, as for example “Morgen komt Piet” and “Demain Pierre vient.”

To summarize it: Under Focus a sentence is limited to five elements; a group can be an element at surface level; within a surface level the order of the elements is free, apart from those instances in which a language uses order to express relations.

**FIGURE 8:**
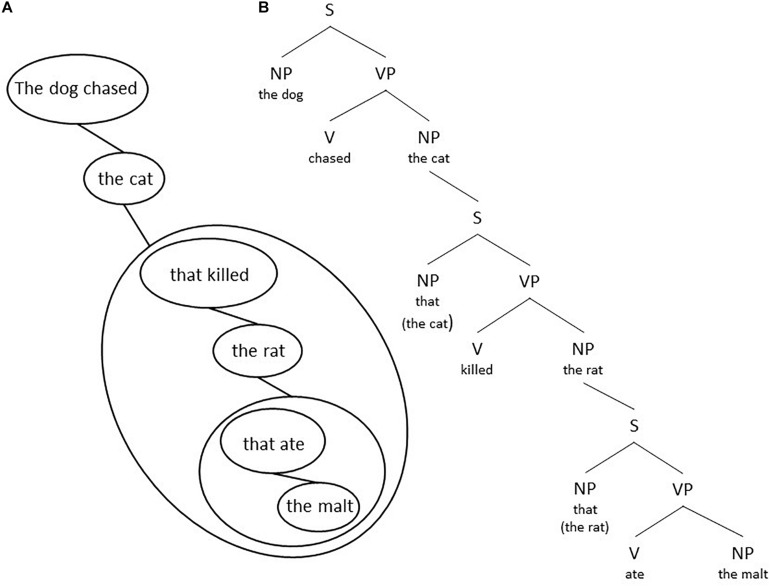
A structural representation of utterance (6b) according to Focus: **(A)** its chunking structure and **(B)** a related grammar structure.

**FIGURE 9:**
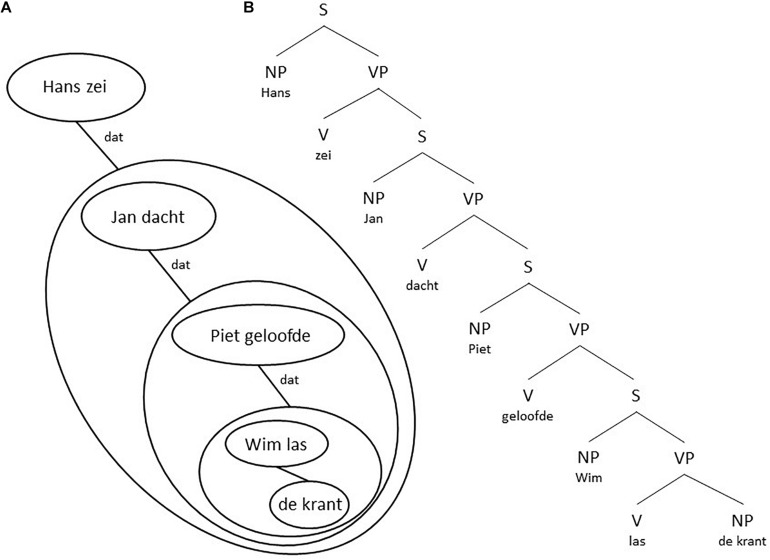
A structural representation of utterance (8a) according to Focus: **(A)** its chunking structure and **(B)** a related grammar structure.

One can make a drawing of the relations between a surface level and its elements. When one starts from the subject and verb in the main sentence and draws the elements and groups down to the right one gets a global structure [see for example Figure 8A for utterance (6b)]. One gets a global grammar structure (see for example Figure 8B) if one uses the standard grammar representation within each group. In a similar way Figure 9 shows the structural representation of utterance (8a). Notice the difference between the grammatical representations in Figures 7, 9B.

If in a language a transposition of a set of elements is allowed, then this property can be used to detect groups. See for example utterances (2) in section “Dual structure in language.” However, the more important point is that Focus theory provides a straightforward answer why these groups exist. A theory of language cannot take their existence for granted. Within a group one can recognize elements and subgroups, but one cannot lift such an element or subgroup out of this group. In sentence (6b) “The dog chased the cat [that killed the rat (that ate the malt)],” both sentences between brackets are adjectival clauses comparable with adjectives. One is a group at the main level, the other one is a subgroup of this group. The sentences in (2) given in section “Dual structure in language” illustrate the classical test case for whether one is dealing with one group or with two different groups. Please recall that sentence (2a) “It is his wife with his new glasses that he sees” is only possible for the sentence in Figure 1B, where “his wife with his new glasses” is one single group. Now let us consider the sentence in (12a).


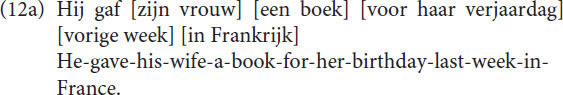


The fact that “vorige week” and “in Frankrijk” are separate groups can be shown by the sentences (12b) and (12c) which can be considered answers to questions like “Wanneer [when] gaf hij etc.” or “Waar [where] gaf hij etc.”


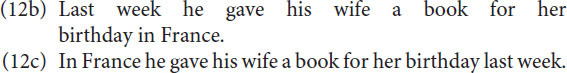


The interesting observation is that according to Focus in (12a) “vorige week in Frankrijk” can be considered a single group as well, which is motivated by the sentences (12d), (12e) and (12f).


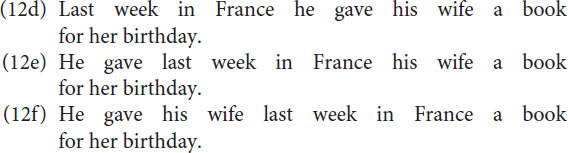


Moreover, the utterances in (12d-f) are not only possible they also seem to be preferred and more natural than (12a), the plausible reason being that it limits the number of groups to 5 instead of 6.

#### Focus and Memory

The theory states that Focus and Memory are distinct cognitive functions. Buffart (2017) suggested that they originate from different types of brain rhythms. A Focus event has a restricted capacity, Memory has not.

Most intended messages have more than one representation. When Memory is involved the elements of an expression are related to each other by means of the various representations of the message. Due to these relations, very complex messages can be communicated, which, due to Focus, almost always have a hierarchical communication structure. One sometimes assumes that the hierarchy in utterances are represented in Memory as well. However, hierarchy does not necessarily exist in Memory. The non-sequential appearance of sentences is due to the Focus limitations, which govern the communication with the outside world, but it does not necessarily mean that Memory operates in this way. In Memory, several connections even between “lower” and “higher” level elements may exist, which indicates that the message is represented by a network, not by a hierarchical structure. Subjects can, for example, easily rearrange words so that a different expression with the same content appears. In language, a high variety of sentences can express the same message. Especially in spoken language each sentence is a rapid evolution of an “accidental” sequence of concepts, depending on the prior concepts and the message intended. All Dutch sentences below, for example, express the same message.


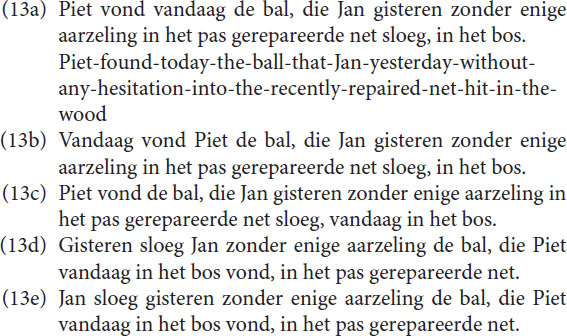



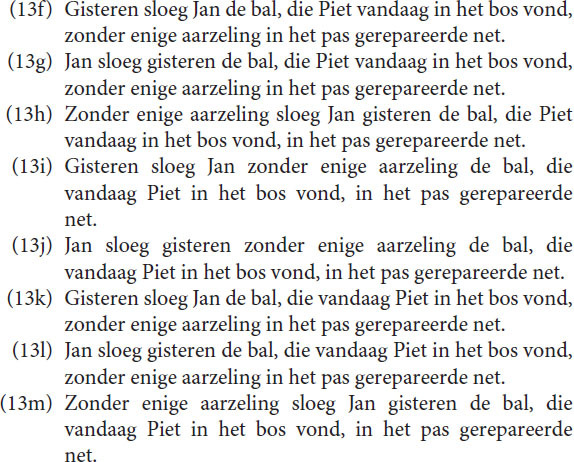


Although each sentence is theoretically correct, and the last five ones require a special emphasis on the word “vandaag,” they are not equally preferred. Preference is a property of Memory, not of Focus. Focus determines the structure. Memory plays a decisive role in the preference for a concrete utterance or perception, involving context and previous experience. We will come back to that in the section “Discussion.”

### Phonology

The arguments why phonological structure exist and why the number 5 turns up there are similar to those we used to explain why Focus forces structures in language. We will not repeat them here.

We will apply Focus-theory to three topics. The first one is the hearing of consonants that are absent in the physical signal. The second one regards the avoidance of interference between the same consonants when in general two words or in particular a word and a suffix are concatenated. The third one concerns the (non-)occurrence of certain shifts of consonants as for example from [k] into [g], from [g] into [ɣ], but not from [k] into [ɣ].

#### Hearing Absent Consonants

One interesting phenomenon is that subjects easily can perceive and produce consonants that are not in the acoustic signal. Listeners can restore missing phonetic segments in words they hear (Warren, 1970) and in speech shadowing experiments (Marslen-Wilson and Welsh, 1978) talkers correct mispronounced words to their correct pronunciation.

As Buffart (2017) argued, a structure that a subject perceives, agrees with the environment when from the subject’s view that structure suffices to cope with in the current situation. Subjects are as accurate as necessary at the very moment, simply because of the fact that the subject actively structures the environment. All oak leaves are similar until one needs the differences. As subjects interpret while listening to (parts of) sentences, they put interpretations on them. When some consonants are missing subjects do not notice their “mistakes” as long as nothing happens that forces a change of interpretation. Notice that this is a nice property, because otherwise we could not talk with each other in a noisy environment.

Completion is not an exclusive language phenomenon. It occurs for all perceptual activities. For example, we use it intensively in vision, when we complete occluded parts of objects. If we couldn’t do that, we would easier loose our bearings in a crowded environment or a wood.

#### Dissimilation as a Consequence of Embeddedness

Dissimilation is known in many languages (Alderete and Frisch, 2007), but we restrict ourselves here to dissimilation in Latin. The standard textbook examples are given in (14).


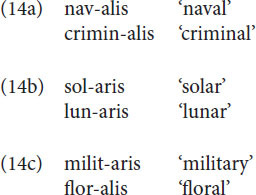


The classical analysis is the one proposed by Steriade (1987). Please recall from section “Structure in language and the magical number 7-2” the different status of liquid sounds like [r] and [l] in French and Korean. In French they are phonemes and in Korean they are allophones. This implies that French speakers need to memorize the feature [+lateral] for [l] and [–lateral] for [r] for word pairs such as *bal*, *bar*; *lire*, *rire*; *bourreau*, *boulot* etc. Korean speakers, on the other hand, do not need the feature [lateral] at all, given that the occurrence of [l] and [r] is entirely predictable, which leads to the famous ‘lice/rice’ problem (Cutler, 2012). In Latin, [l] and [r] are phonemes as well (*rex* ‘king’ vs. *lex* ‘law’). Steriade assumes that only sounds for which the feature [lateral] is relevant are specified for that feature. For the Latin coronal consonants [t,d,s,n,l,r] the feature is only relevant for the last two sounds, the group of [CORONAL, +approximant] sounds (cf. Figure 4 in section “Structure in language and the magical number 7-2”), and is also the only feature that contrasts these two sounds. On the assumption that [t,d,s,n], the group of [CORONAL, -approximant] sounds, are not specified or unspecified for [lateral] and that this group has a redundant, surface, [–lateral] specification, Steriade accounts for the dissimilation effects in (14) as follows. The underlying form of the suffix is assumed to be -alis as in (14a). In (14b) [l] changes to [r] if the base contains an [l]. However, if between the [l] in the base and the [l] in the suffix an [r] intervenes, dissimilation is blocked, as in (14c). Please observe that although the intervening [n] and the intervening [t], in *lunaris* and *militaris*, are just as [r], [–lateral], they do not block dissimilation. Steriade’s explanation for this is that the feature [–lateral] is redundant for the [t,d,s,n] group, but contrastive for the [r,l] group.

Cser (2010), referring to Dressler (1971) and Hurch (1991), has claimed that not only an intervening [r] blocks dissimilation, but that intervening labial and dorsal consonants block dissimilation as well. Here a word of caution on the data is in order. The data given in Dressler (1971) go back to von Paucker (1885) who compiled lists of words with and without dissimilation, without taking into account the moment in time the word occurred. So, a form like *palmalis* is given as a double form for *palmaris* ‘of palms.’ The form *palmalis* does occur (Library of Latin texts A and B^6^), but is a hapax as it occurs only once at around 1050 (Petrus Damiani).

In Table 1 we listed all words with an *l* in the stem and *alis* or *aris* as a suffix that we found by checking the A and B corpora of Latin texts on www.brepolis.net (more than 60 million forms) including all late forms up to the Middle Ages. It shows that besides an intervening [r], intervening dorsal consonants, [k] and [g], block dissimilation as well, as illustrated in Table 1A. There are three exceptions to be noted: *culicaris*, *vulgaris* ‘of the mass’ and *caligaris*, ‘of the boot.’ The form *culicaris* does not occur in the A or B corpus, but does occur in the Brepolis database of Latin dictionaries in four dictionaries, once it is given as *culicaris* an adjective *ad culices pertinens* ‘pertaining to flies,’ but three times as a noun *culiculare* ‘mosquito net.’ The form *caligaris* occurs nine times in the A corpus, five times before 500 AD and four times after 500 AD. The form *vulgaris* is common in A and B and occurs only once in A as *vulgalis* (Thomas de Aquino). The dictionaries give for the last two forms alternative forms with the same meaning *caligarius* and *vulgarius* which might explain the non-blocking in the related forms *caligaris* and *vulgaris*.

**TABLE 1:** **(A)** suffix, *alis* only; **(B)** suffix *aris* only; **(C)** suffixes *alts* and *aris.*

**(A)**						

Blocking interference		Words	Compiled words			
dorsal consonants	7	legalis, glacialis, umbilicalis, intellectualis, vulcanalis, localis, lecticalis				
palatal glide [j]	2	caelestialis, pestilentialis				
r	6	floralis, littoralis, lateralis, pluralis, liberalis, larvalis (4 × between 200 and 500 and 17x after 500) = larbalis (4x after 736)				
*fl or gl*	5	fluvialis, flavialis, glebalis, flaminalis	con′gluvialis			
no/in second part	1		sol’stitialis			
no explanation	1	embolismalis	sm/m?			
Total	22					

**(B)**						

**Interference**		**Words**	**Explanation**			

single l	9	lunaris, solaris, militaris, ridicularis, lupanaris, liminaris, lapidaris, lupinaris, limitaris				

*cl*	1	Tricliniaris				
dorsal: explanation	2	caligaris, culicaris	(cali)(gali)s, (culi)(cali)s			
dorsal: no explanation	1	Vulgaris				
Total	13					

**(C)**						

**Words**	***aris***			***alis***		
**10**	**Before 500**	**After 500**	**Reason**	**Before 500**	**After 500**	**Reason**

latia*is	30	13	/	3	10	palatal glide [j]
linea*is	12	27	/	1	43	palatal glide [j]
proelia*is	×		/		×	palatal glide [j]
pluvia*is		1	/	42-n	n	palatal glide [j]/*pl?*
elementa*is	×		/		×	No explanation/*m*?
lumina*is	3	44	/		5	no explanation/*m*?
fulmina*is		3	/		2	*lm/m?*
palma*is	5	2	/	0	1	*lm/m?*
cloaca*is		5	/	1		dorsal *c*
lectua*is		1	/	2	9	dorsal *c*

* means l or r.

We cannot support Cser’s claim that labial consonants, [b], [m], [p], [f] block the dissimilation as well. There is no blocking in *liminaris* ‘of a threshold,’ *limitaris* ‘of/on the border,’ *lupanaris* ‘of a lewd woman,’ *lapidaris* ‘of stone’ and *lupinaris* ‘of a lupin seed.’ The apparent exceptions to the general finding that intervening labials do not block dissimilation, that is words like *fulminalis* ‘of lightning,’ *elementalis* ‘elementary,’ (*annus*) *embolismalis* ‘year of 13 lunar months,’ *luminalis* ‘of light,’ *limitalis* and *palmalis* are all later forms and occur after the 5th centuray AD. We only found the glide [w] as an instance of a blocking labial segment. However, the Latin [w] was a labio-dorsal glide, so by having a dorsal articulator as well, [w] is expected to pattern with the dorsal consonants in blocking the dissimilation and one may thus understand the blocking in *fluvialis*, *pluvialis*, and *flavialis* (Table 1A).

The vowels [e] and [i] became a glide [j] in the 1st century, which thereby became a consonant (acquiring a C-place in Clements and Hume, 1995) and which also has a dorsal articulator, and one may argue that in a similar way to the glide [w], the later glide [j] in the first three forms in Table 1C started to block dissimilation (*proeliaris*, *proelialis*, *linearis*, *linealis* etc.). There is only one real exception to the observation that labial consonants do not block dissimilation and that is the word *glebalis* ‘of clods.’ The classical form of the word is *glaebalis* and, again, given that –*ae*- [aj] is a vowel-glide sequence, the [j] may explain the blocking.

Focus theory learns that if in a sequence two non-neighboring elements are related, the elements in between are grouped or embedded. So, in GKLGHJ K and L are embedded G(KL)GHJ. In this representation G(KL)G is a part in itself and so is HJ: G(KL)G/HJ. If this is not the case and if the sequence has to be seen as a unity, the better representation is G(KL)G(HJ), expressing the fact that G is not the end of the first part, but rather the beginning of the second part. This can be clearly recognized in longer expressions like G(KL)G(HI)G(JM)GN.

Latin liquid dissimilation can be understood by this property. We like to concentrate on the question why dissimilation occurs and what blocking means. The suffix *alis* creates a problem for *luna*, since according to Focus the embedding *l(una)l/is* occurs, breaking the word *luna* and the suffix *alis* as well. For the same reason Focus theory learns that the suffix of *flora* is *alis* and not *aris* since the *r* in *floraris* would interfere in a similar way as the *l* in *lunalis*, whereas the common assumption on dissimilation assumes that the *r* in *flora* blocks the dissimilation. In general, there must be a clear distinction between the stem and the suffix.

We thus assume that dissimilation only occurs, when due to interference embedding or grouping takes place that mixes a part of the stem and a part of the suffix. We found several reasons while interference does not take place as for example a dorsal consonant between both *l*, or the fact that a stem is a compound word with the *l* not in the last part^7^. As mentioned above, there are two apparent exceptions of the blocking by dorsal consonants, *culicaris* and *caligaris* (see Table 1B). These are interesting, because in our analysis these are not exceptions since the interference is not an interference between *l* but between more complex sound combinations, possibly including the dorsal consonants. The combinations *culicalis* and *caligalis* are both impossible, at first sight because of the groupings *cu(li)(ca)(li)s* and *c(ali)g(ali)s*, but more probably because of *(culi)(cali)s* and *(cali)(gali)s* which are groupings when one introduces functions, (*c?li*) and (*?ali*).

We argued that in *fluvialis*, *pluvialis* and *flavialis* (Table 1A), the glide [w] blocks as well, and that in a similar way glide [j] blocks in *glebalis* since it originates from *glejbalis*. However, from the viewpoint of interference there might be a different explanation, holding that the *fl* en *pl* (see Tables 1A,C) do not interfere with a pure *l*. This could also explain the existence of *congluvialis* and *flaminalis*. Two other apparent exceptions in Table 1A are compound words since they have no *l* in the second word part.

*Esbolismalis* is an exception that we cannot explain; several interferences (*l*, *li*, *lis*) are possible. The only explanation is that *lism* was pronounced as strong unity. It should also be observed that it is a late form. In the Library of Latin texts A corpus, it occurs once before 735 and 74 times after 735. In the course of ages, after 500 AD, *aris* became an accepted alternative to *alis* (see Table 1C), but not for the cases where interference would occur, as *floraris*. Notice, that the revers took place as well; *luminaris* has an alternative form *luminalis*, but just as *embolismalis* it occurred after 500 AD, so that it might be an artifact influenced by the current languages spoken at that time.

The idea is that dissimilation only occurs, when due to interference embedding takes place that mixes a part of the stem and a part of the suffix. We will not pursue this issue further here, but just note that it would be interesting to investigate dissimilation in other languages from this interference viewpoint.

#### Consonant Shift

It is typologically quite common to observe in languages that in an intervocalic position consonants tend to weaken. Voiceless consonants become pronounced as voiced, a modification known as VOICING and voiced plosive consonants become pronounced as voiced fricative ones, generally termed SPIRANTIZATION. In Table 2 we give some relevant examples from Sisco Corsican (Cravens, 2000; Gurevich, 2004) and from Gran Canarian Spanish (Oftedal, 1985). In Table 2A for example, the voiced plosives are realized as voiced fricatives and the voiceless plosives as voiced plosives when occurring in intervocalic position.

**TABLE 2:** Examples of consonant shifts.

(A)	Corsican voicing of voiceless consonants					
	**voiceless**			**voiced**		
1	[p] [pane]	‘bread’		[b] [ubane]	‘the bread’	
2	[t] [tεmpu]	‘time’		[d] [udεmpu]	‘the time’	
3	[k];kane]	‘dog’		[g] [ugane]	‘the dog’	
**Corsican spirantization of voiced plosive consonants**						
	**Voiced plosive**			**voiced fricative**		
4	[b] [bok:a]	‘mouth’		[β] [aβok:a]	‘the mouth’	
5	[d] [dεntu]	‘tooth’		[ð] [uðεnte]	‘the tooth’	
6	[g] [gola]	‘throat’		[γ] [aγola]	‘the throat’	

**(B)**	**Canarian**	**Spanish**		**Canarian**	**Spanish**	

**Voicing of voiceless consonants**						
	**Voiced**	**Voiceless**		**Voiced**	**Voiceless**	
1	[g] [tibigo]	[k] tipico	‘typical’	[b] [unabluma]	[p] [una pluma]	‘a feather’
2	[d] [frudero]	[t] frutero	‘fruit bowl’	[d] [unadjenda]	[t] [una tienda]	‘a shop’
3	[g] [musiga]	*[k]* musica	‘music’	[g] [lagama]	[!k] [la cama]	‘the bed’
4	[ 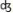 ] [fle 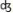 a]	[ 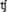 ] flecha	‘arrow’	[ 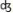 ] [una 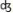 iga]	[ 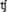 ] [una chica]	‘a girl’
**Spirantization of voiced plosive consonants**						
5	[ð] [naða]	[ð] nada	‘nothing’	[ð] laðoma]	[ð] la doma	‘the taming’
6	[β] [roβa]	[β] roba	‘steals’	[β] [laβaxa]	[β] la baja	‘the
7	[γ] [plaγa]	[γ] Plaga	‘plague’	[γ] [laγama]	[γ] la gama	‘the range’

**(C)**	**Surface contrasts in Gran Canarian Spanish**					

	**Canarian**	**Spanish**		**Canarian**	**Spanish**	
1	[d] [nada]	[t] nata	‘cream’	[ð] [naða]	[ð] nada	‘nothing’
2	[b] [roba]	[p] ropa	‘clothes’	[βl [roβa]	[β] roba	‘steals’
3	[g] [plaga]	[k] placa	‘plate’	[γ] [plaγa]	[γ] Plaga	‘plague’

The interaction between VOICING and SPIRANTIZATION that can be observed in Table 2 is extremely hard to describe and to explain by current phonological theory. There have been two main ways of describing the relation between stored, underlying forms of words in the mental lexicon and their concrete realization in speech. The Corsican words for ‘bread,’ ‘time’ and ‘dog’ in (2a) are commonly assumed to be stored in memory with an initial [p], [t], and [k]. Their surface realization after a vowel as [b], [d], and [g] is assumed to be the result of a phonological modification that takes place when a speakers picks up words from memory and combines them into sentences. The dominant first view in generative phonology is to consider these modifications as a series of ordered rules. The way in which the two rules of VOICING and SPIRANTIZATION have to be ordered for the data in (2) is in such a way that VOICING applies first, so that it is no longer applicable after SPIRANTIZATION has been applied. This order, however, is considered a problematic one, given that it is not clear why, if there is VOICING, VOICING does not also affect the outcome of SPIRANTIZATION. The assumption (Hale and Reiss, 2008) is that this order should be the dispreferred one in languages. However, Gurevich (2004) has shown for 230 voicing and spirantization processes in 153 languages that in the majority (92%) of cases, this is, contrary to expectation, exactly the order that one finds. For the second dominant view in generative phonology, Optimality Theory, the data in (2) are even more problematic. The assumption of Optimality Theory is that stored words are evaluated with respect to a number of output well-formedness conditions, one of which would say that a voiced consonant in intervocalic position should be pronounced as a fricative. Although this accounts for the modification of the initial [b], [d], and [g] cases in (2a; 4–6) it fails for the initial [p], [t] and [k] in (2a; 1–3). Why is there still a surface intervocalic [b], [d], [g], if there is a well-formedness condition requiring that they should be pronounced as fricatives? We refer for further critical discussions of various proposals that have been put forward within the constrained-based framework to Jacobs (2019).

What the rule-based and the constraint-based models share is that they both are speaker-oriented, that is, they model the connection between the stored, underlying word form, say *la gama*, and the actual pronunciation of that word by a speaker, [laama], without looking at the perception-side, where the listener has to interpret the heard form, say [laama], as being the stored, underlying word form, *la gama*.

We think that Focus-theory also might provide a new way of looking at phonological modifications that have proven to be problematic for these production-based models, which are processing models. Focus-theory states that not processes, but representations are the key; these are dictated by Focus. We will explain the phenomena on the basis of “the other side of the coin,” which means not on the maximum length of expressions but on the existence of two complementary representations in Focus. The research question then is what do these representations look like.

**FIGURE 10:**
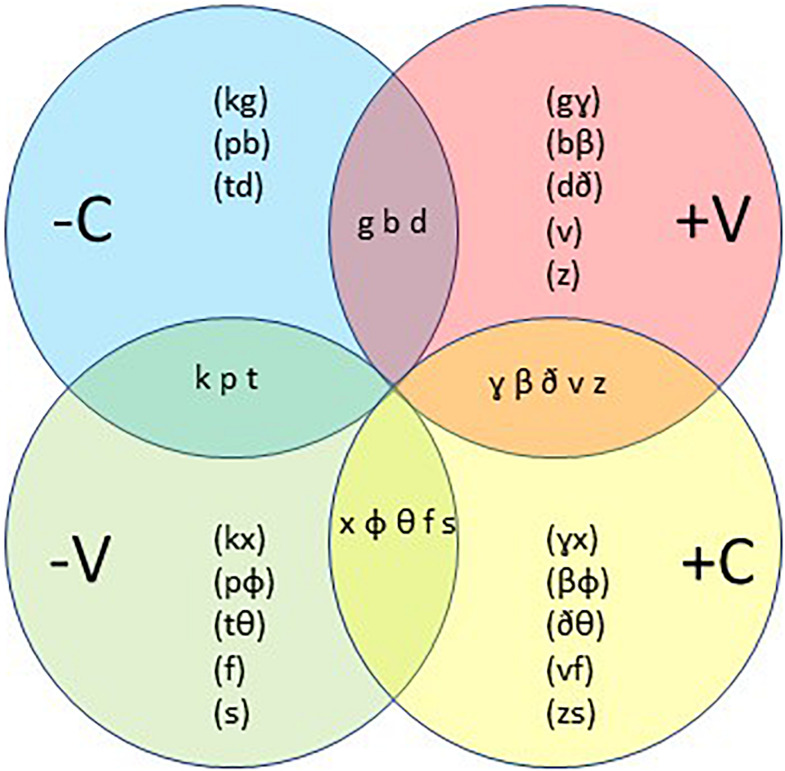
Representations of consonants. The circles refer to representations, their intersections to the physical utterances.

The consonants [k] and [g] have the same features except that the voice feature of [k] is negative (–V, voiceless) and the voice feature of [g] is positive (+V, voiced). In a similar way [g] and [ɣ] only have a different continuant; [g] has a negative or plosive (–C) feature and [ɣ] has a positive, fricative or continuous (+C) feature. Figure 10 shows in each circle mostly pairs of consonants. Each pair either agrees on the V feature and differs on the C feature or agrees on the C feature and differs on the V feature. For each pair, all other features are equal, which means that the members of a pair are produced in the same way except for the V or the C feature. In the overlaps between the circles one sees the consonants with both the V and the C specified. The way of presentation in Figure 10 is not arbitrarily chosen. We think that it will be helpful to explain how, according to Focus-theory, the consonants may be represented in the cognitive brain.

We assume that not the specific consonant is stored in memory, but rather the representations of the features for its pronunciation, which trigger the sensorimotor control of the pronunciation. The consonants in a trio like [k], [g], [ɣ] have a lot of features in common. Since all features for these three consonants are the same except V or C, we concentrate on these two. [k], [g], [ɣ] only differ in a combination of them: plosive (–C) or fricative (+C) and voiced (+V) or voiceless (–V). So, [k] has the features [–C–V], [g] [–C+V], [ɣ] [+C+V] and we can add to these [x] [+C–V]. Assuming that not the pronunciation itself is stored, but the representations that trigger the moving forces behind, we find that –C implies [k] and [g], +C implies [ɣ] and [x], +V implies [g] and [ɣ], and –V implies [k] and [x]. Notice that in –C, the V feature is not fixed, or in other words, the –C representation of [k] or [g] is ambiguous with respect to V. This holds equally for +V (ambiguous with respect to C), +C and –V. The representations –C and +C on the one side and –V and +V on the other are complementary to each other. This can also be read in Figure 10 in the sense that one finds [k], [g], [ɣ], and [x] in the intersections of C and V.


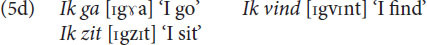


Whether a –C consonant will become [k] or [g] depends on the context. In accordance with section “Structure in language and the magical number 7-2” (Figure 4) shifts can only take place within one representation. To elucidate: in the +C representation the V can move between +V ([ɣ]) and --V ([x]) but cannot between --C and +C since +C is fixed. This explains why [k] can shift to [g], [g] can shift to [ɣ], but there is no shift from [k] to [ɣ], and vice versa^8^, which is precisely what Sisco Corsican and Gran Canarian Spanish show in Table 2. In a –V context, as for example in [kane] (A3), the pronunciation will be as a [k] and in a +V context, as in [ugane] it will be as a [g]. This similarly holds for +V, where the pronunciation will be [g] in a –C context, as in [gola] (A6) and a [ɣ] in a +C context, as in [aola]. Here the silence before a word is a [–V–C] environment and a vowel is a [+V+C] environment. The other consonants in Figure 10 behave in a similar way, such as for instance the trio [p], [b], [β], as in [roba], ropa, [roβa] and roba (C2), and the trio [t], [d], [ð], as in [nada], nata, [naða] and nada (C1). Other examples of shifts allowed by the model are [pane] and [ubane] (A1), [tεmpu] and [udεmpu] (A2), and [baxa] and [laβaxa] (B6).

We will also use this model to explain the shifts in (5). So, we assume that for each consonant in a word one of the features is determined, either the C feature or the V feature. The other feature is determined by the context during the utterance. Moreover, in Dutch the ending consonant of a word is ambiguous with respect to voice. This means that during speech production and perception voice or no-voice is easily influenced by the context.


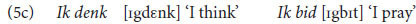


So, the *k* in *Ik* in (5c) has a –C feature and is ambiguous with respect to V, and the *d* and *b* in *denk* and *bid* in (5c) have a +V feature and are ambiguous with respect to C. Therefore, the only adaptation possible is that [k] becomes +V, which means [g]. In short, [**–C**–V]/[–C**+V**] → [**–C**+V]/[–C**+V**]. This regressive assimilation often occurs in French since many words begin with a +V feature fixed; for example, the order *vowel-p-v-vowel* becomes in French *vowel-b-v-vowel* but in Dutch *vowel-p-f-vowel*, a progressive assimilation due to the –V property of the consonant at the end of a word.

The *k* in *Ik* in (5b) has a –C feature and is ambiguous with respect to V, and the *g*, *v* and *z* in *ga*, *vind* en *zit* in (5b) have a +C feature and are ambiguous with respect to V.


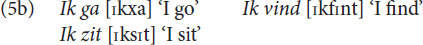


Our model delivers two solutions. First, the progressive voice assimilation showed in (5b). Here the second consonant changed from [ɣ], [v] and [z] into [x], [f] and [s]. In short, [**–C**–V]/[**+C**+V] → [**–C**–V]/[**+C**–V]. The second solution is an adaptation from [k] in *Ik* to [g] as shown in (5d). In short, [**–C**–V]/[**+C**+V] → [**–C**+V]/[**+C**+V], a regressive voice assimilation.

Some 6% of the Dutchmen, living in the south of the Province Limburg, prefer the second solution (5d). It might be tempting to try and explain why Limburgian Dutch and French would have a preference for regressive rather than progressive assimilation. It could be related to the frequency of occurrence of phonemically contrastive voiced and voiceless fricatives, rather limited in Standard Dutch, rather absent in Randstad Dutch, less so in Limburgian Dutch and frequent in French. Alternatively, melody and tone might play a role. We will not venture a further explanation here.

The study of Gurevich (2004), mentioned above, suggests that the model represented in Figure 10 may cover 92% of the investigated languages in her corpus. The model completely fits with the main theorem of Focus-theory, which implies a maximum of two representations in Focus. Since Focus governs the communication with the environment, this model holds for production and for perception as well. The latter is in line with the assumption that the human system is an active system that always operates on the environment. The model explains which shifts are possible. Shifts between [k] and [g] are allowed, when the consonant in the word is memorized as –C, whereas shifts between [g] and [ɣ] are allowed, when a consonant is memorized as +V. The model represented in Figure 10 obviously only holds for languages where the differences between consonants are based on the properties fricative/plosive and voiced/voiceless, that is on the features ‘continuant’ and ‘voice.’ If that is not the case, as for instance in Korean, which does not have a phonemic +V/–V difference, shifts do not occur, or other shifts may take place. However, the prediction is that whatever the model for a given language will be, it will always be based on two complementary representations.

In Dutch [v], [z], [ɣ] form the [+V+C] group and [b], [d] the [+V–C] group. The [–V+C] and [–V–C] groups are [f], [s], [x], and [p], [t] respectively. In Dutch the consonant at the end of a word is –V, so the model in Figure 10 may help to understand the pronunciation of some verbs in section “Where does structure come from?” that are voiced in their plural form but voiceless in their singular form like *wij geven* and *ik geef*, *wij lezen* and *ik lees*, *wij zeggen* and *ik zeg, wij hebben* and *ik heb* and *wij wedden* and *ik wed*. The pronunciation shifts are from [+V+C] to [–V+C]: *geven* [v] → [f] *geef*, *lezen* [z] → [s] *lees*, *zeggen* [ɣ] → [x] *zeg* and from [+V–C] to [–V–C]: *hebben* [b] → [p] *heb*, *wedden* [d] → [t] *wed*. In (15b) one can see that in *hebben*, the first person singular form shows regressive voice assimilation. In section “Where does structure come from?” we also showed that there are verbs that are voiceless in both plural and singular form, as *kopen* and *wensen*. These verbs also show in the first person singular regressive assimilation to voiced as shown in (15b) and (15e). The voiceless pronunciation at the end of Dutch words is shown in (15a) and (15d). This also occurs in (15c) and (15f) since the following word starts with a voiceless consonant.


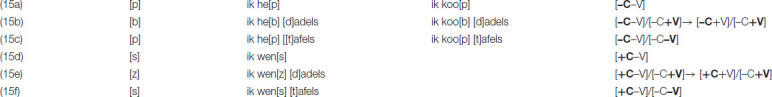


## Discussion

The theory used here is a theory on cognition that rests on three principles: first, the human cognitive system is an active system that focuses, which means: it does not processes information but it creates the information in interaction with its surroundings; second, this interaction is based on sequences; and third in a representation different types of relations between elements do not interfere. The consequence is the connection between 5 up to 7 elements in Focus and the use of maximally two complementary representations in Focus. These are two sides of the same coin. This mathematical result convinced us that the overwhelming occurrence of 7-2 in psychology and language is not a pernicious Pythagorean coincidence, but a characteristic feature of human cognition.

Focus is the intermediary between Memory and the surroundings. This surroundings is fluid. It is not only outside the body, but can also be thoughts, images, concepts, percepts et cetera, or in other words, each content in the brain. Focus structures^9^ the surroundings, which is only possible if Focus relies on a general brain property. Therefore Buffart (2017) suggested that cross frequency couplings of local brain rhythms may determine Focus and that global couplings may determine the activation level of the other cognitive operations, like Memory, and for that reason, preference and attention. This implies that local rhythms determine the structure and that global ones between brain regions determine the strength of the relations between these structures. The latter system requires a broadly branched network since it combines relations to all senses and the effectors of the body. If the suggestion is correct individual differences in Focus-capacity could also be found by differences in cross frequency couplings of local brain rhythms.

Davis and Holmes (2005) reported on experiments in which they showed that the number of objects in a visual short-term memory (VSTM) experiment is not a fixed number of 4 contrary to what Luck and Vogel (1997) have claimed. Davis and Holmes constructed stimuli with four clusters of visual objects, each having a colored small left and right part and a white circle or oval in between. When the left and right parts attach the circle or oval, they are together seen as one single object, partly occluded by the circle or oval. When they do not attach, both parts are perceived as two objects. In relation to Focus, the fifth experiment that Davis and Holmes reported on, is the most interesting. Here, the left part is the mirror image of the right part. The difference between the results of this experiment and those of the other experiments can only be understood, if one assumes that subjects have noticed the symmetry.

Since according to Focus theory, form detection occurs within Focus, processing in Focus must take place before, or simultaneously with, STM processing. Let us therefore have a closer look at one of the experiments described in Cowan (2000). Subjects hear numbers, while they search on a computer for words in four surrounding fields that rhyme with a word in the central field. After some time, the inputs stop and subjects have to recall the numbers in the right reversed order, beginning with the last one. The error rate is measured, showing a mean low rate between 2.5 and 3.5 numbers and a low rate for individual differences between 2 and 6.

When the numbers pass Focus, chunking is necessary to handle the stream of numbers. Let C5 mean a chunk of span 5. Then for Focus span 7, one can represent the row by the chunks C5C2 and its complementary representation C4C3^10^. Both together cover the row. In symbols (ABCDE)(FG) and (ABCD)(EFG), where A refers to the last number^11^. For Focus span 7, C5 and C4 are the relevant chunks, for span 6, C4 and C3, and, for span 5, C3 and C2. Since there is no preference for one of the representations, the mean chunk lengths are 4.5, 3.5, and 2.5 respectively, and individual differences may vary between 2 and 5, both in line with the findings of Cowan (2000). So, Focus theory is in line with the experimental results, but the interpretation is different given that Focus does not use a limited memory concept. The suggestion of a limited memory comes from the fact that unrelated elements pass Focus, which has a limited capacity and builds chunks in order to cope with them. Focus “catches” the input, using maximally two representations, each obeying the non-interference between both types of possible relations between its elements.

Given that in perception, the outcome of Focus is determined by a combination of input from the sensory register and from long-term memory the question arises, whether there is a relation with the concept of working memory (WM). Focus does not describe any processing as WM does (Baddeley, 2000). If, according to Baddeley, WM is an LTM-independent mechanism, there might be a relation between Focus and WM, and Focus would describe the structure of the results of its processes. If not, WM is part of Memory. Focus and Memory are distinct cognitive functions. Processing occurs in Memory, and, as far as this theory is concerned, is restricted to preference. As Baddeley et al. (2019) admit the question whether the processes assigned to WM could be part of the long-term system, is still an open one. However, that may be, Focus plays a role in the intermediate stage between memory and sensory and equilibrium organs, but also plays a role in thinking, when one thought is related to another one, and in production as well, when subjects respond or produce other utterances. The restrictions come from Focus, not from Memory.

From this it is clear, that there is some relation between Focus and Attention, but these concepts are not identical. They rest on different theoretical premisses. Attention, and in the same way Working Memory, are concepts in the tradition of information processing. The information comes from the environment, feelings and thoughts of the subject. Focus theory takes the subject as an active system, for which a hypothesis testing system could be used as a metaphor. Marchetti (2014) regards episodic memory and episodic future as constructive processes. He states “Regarding the construction processes underpinning the various forms of consciousness, we have seen that the basic elements are provided by attention, and that their assembly is ensured by working memory.” Oberauer (2019) discussed control mechanisms in the interaction between Working Memory (WM) and Attention and concluded that “WM plays a crucial role in controlling attention and action by holding the representations that guide attention and action. The control process consists of selecting these representations into WM – once they are established in WM, they have their influence on attention and action automatically …”. These two views are very close to the basic concepts of the Focus mechanism. Focus theory supposes that representations which make up the hypothesis of the current environment/surroundings (cf. fn. 5) are more activated and thus, one could say, embody a “temporal working memory.” The most activated representation and in most cases a complementary one appear in Focus. If (a part of) the Focus content does not fit with the environment/surroundings, new representations appear by a change of the activation field. Although Focus and Attention are different concepts, the most activated representation in Focus can be seen as the “focus of attention” (Oberauer, 2019). Notice that, in the information processing model, a top down controlling mechanism for Attention has been introduced, whereas in Focus this controlling mechanism is implicit.

Although Focus and Memory are distinct cognitive functions, the differences in Focus-capacity lead to differences in Memory. There are 16 possible representations for Focus-capacity 5, 43 for Focus-capacity 6 and 163 for Focus-capacity 7. Most representations could be associated with more than one pair of complementary representations. Therefore, the number of potential and actual associations in Memory rapidly increases with Focus-capacity, which in turn leads to richer memory networks^12^. In general, it is hard to show directly that structures in Focus determine the associations in Memory. But experiments with serial patterns showed (van Leeuwen et al., 1988; van der Vegt et al., 1989; van Leeuwen and Buffart, 1989) that the idea that structural interpretations of stimuli govern the network structures in memory (Buffart, 1986) makes sense. Serial patterns were memorized, for instance, in terms of their structural components, and associations between them were based on complementarity between their representations. Although Focus operates sequentially, the finite number of possible sequence structures guarantees that the processing of the system is fast. With respect to this the findings of Elliott and Giersch (2016) are interesting. These show that in a “psychological moment” of at most 50–60 ms the elements are processed successively. Moreover, assuming that a basic processing unit takes 4.5 ms (Geißler, 2018) for Focus-capacity 7, one could roughly say that for an experienced reader a linear sentence as (3) “Nous avons appris le français grâce à nos enseignants” would roughly take 1.5 s, being 7^∗^4.5 ms (≈31.5 ms) for a syllable, 7^∗^7^∗^4.5 ms (≈ 220 ms) for the phonological phrase and 7^∗^7^∗^7^∗^4.5 for the sentence.

The theory presupposes that Focus processes sequences. This means that each communication between the mind and its surroundings is sequential in principle. But theories which advocate hierarchy in human communication have a point as well. Due to the Focus limitations every expression and every percept have at the surface level a limited number of elements that are processed at once. But these elements are mostly very complex expressions themselves or in the case of language often “refer to” complex objects or situations. In languages one easily recognizes this hierarchy, as for example, in subordinate clauses, a grouping of adjectives and a noun, and the limited number of distinct elements in sentence parsing.

Focus forces hierarchy, but it does not prescribe which structure is preferred. It only learns that within one representation differing relations between elements exclude each other, and that there are maximal two representations to compose an expression for production or perception. Some researchers suppose that humans have a special inborn ability for language grammar or for its building blocks. There is no need for such an assumption since the limitations of Focus force hierarchical constructs and, on the other hand, set restrictions to the depth of these constructs.

The Focus limitations not only generate hierarchy in language, they also generate the perception of visual form and meter in music as shown in the work of the Structural Information Group in Nijmegen (Leeuwenberg, 1971; Collard et al., 1981; Buffart and Leeuwenberg, 1983; Leeuwenberg and van der Helm, 2015) and they appeared to be applicable to explain reaction times in verification and classification tasks of objects according conceptual categories (Buffart and Geißler, 1984; Geißler and Buffart, 1985). All experienced structure is generated by Focus. For sure, pre-structured input will be helpful to detect structure, but this does not mean that experienced structure is only usage-based as, for example, Bod (2002) assumes. When several structures are possible, usage may influence the preference for some structures above others, but not structures as such. According to Buffart (2017) preference is not a Focus issue. Focus does not determine the preference for some language expressions above others or for some interpretations of visual objects above others. Focus governs structure, but preference is governed by Memory. Intelligence and creativity are based on Focus not on Memory. This implies that the nature of human intelligence fundamentally differs from computer intelligence.

Memory is a type of neural network or connectionist model. It is a network of representations, that is based on relations between their elements. The more activated relations a set of elements has, the stronger it is. In principle the more preferred representation is the representation with more and stronger activated relations. This depends on

• the internal structure of a representation composed of type A and type B relations. Without any context or memory influence, one can predict which representations will be preferred. A representation with more structure has less degrees of freedom, which is known as the minimum principle, and is therefore stronger activated in isolation. In visual perception research, it is easier than in language experiments, to present stimuli without context and thus in isolation, and indeed, then the predictions on preference are impressive (Leeuwenberg and Buffart, 1983). In language experiments context and memory always will play a role.
• the context, which might be context within the message itself or from the circumstances as, for example, a preceding question or discussion, or even an experimental task (Buffart et al., 1981).
• memory. It influences the syntactical correctness of sentences. As far as it is not a consequence of Focus, correctness is usage-based. Although the order of the elements in a Focus representation is free, a word sequence, in which the words that are directly related to each other are neighbors, will have representations with more relations than sequences, in which they are not neighbors. In this sense the interaction between preference and Focus directly influences order. Notice that due to the general activity principle of the theory, Memory is an always active network and that the more activated representation and its more activated complement are the representations that are active in Focus. In consequence of memory influence, they are not necessarily the representations with less degrees of freedom in isolation.

For research it is nice when one could present stimuli in splendid isolation, since then representations are characterized by the minimal degrees of freedom, which generally in daily life do not occur. We emphasize that this minimum is not a process criterium as Chater (1996) and Bod (2002) suppose. Focus-theory is not a process theory. On the contrary, it tells that one first must understand structure (Focus) in order to be able to understand processing (Memory). For sure, there is interaction between structure and processing in the sense that the most preferred structures in Memory determine the Focus representations and that the representation in Focus induces Memory activity. The outcomes are preferred representations. In the interaction with the environment one might better consider it as stability, which includes not only the influence of context and memory as is almost always the case in language but also the extent of the analyses of the environment as far as momentarily necessary for the subject.

An active cognitive system implies that perception is silent production. Language perception and production are active processes which generate structure. The fact that this structure is often similar for many languages does not imply that this has to be attributed to a genetical blueprint, but rather to the overall working of the active brain. Some research directly supports the idea of an active brain. Bolton (1894) showed that physically identical sounds made by an electronic metronome are nevertheless perceived by listeners as auditory grouped. Iversen et al. (2008) report that Japanese and English participants showed different patterns of perceptual grouping of sequences of tones in which every second tone had either increased amplitude or increased duration relative to the first tone. These differences are attributed to different native language influence. Other support for the notion of an active brain is presented by neurologists (De Ridder et al., 2015) to explain tinnitus and other disorders.

For us the more defiant research question is to show that one can apply the general Focus-theory to explain a variety of language phenomena, knowing that the theory has already been applied successfully to visual form perception. In other words, did we find a more general approach to structural aspects of cognition? The theory does not only describe how languages and phonology behave, but much more why they do so. Some phenomena are of general nature as the tension between the sequential and the hierarchical character of language. It, however, can also account for the perceptual limits on the number of embedded sentences. It makes clear why the number 5 so often turns up in language and phonetics research. We used the other side of the coin, two complementary representations, to build a model explaining the properties of consonant shifts. This notion of complementary representations opens possibilities for research. Imagine, for example, that there is a representation of the elements of the message and a representation of the modus of the message. In this way one might understand the difference between “John falls in love with Mary” and “Falls John in love with Mary?”. In the latter sentence the question mode is expressed by the inversed order of the subject and the verb. That is not always the case. The question mode can also be expressed by the intonation or music of the sentence (“John falls in love with Mary?”). There is a big research challenge to look at language phenomena from such a complementarity viewpoint.

## Data Availability Statement

The original contributions presented in the study are included in the article/supplementary material, further inquiries can be directed to the corresponding author/s.

## Author Contributions

Both authors listed have made a substantial, direct and intellectual contribution to the work, and approved it for publication.

## Conflict of Interest

The authors declare that the research was conducted in the absence of any commercial or financial relationships that could be construed as a potential conflict of interest.

## Footnotes

1 In languages such as Dutch, English or German there is an intermediate structure between the syllable and the phonological phrase, where syllables are grouped in binary feet. A phonological phrase or accentual group can therefore contain more than seven syllables. A German compound word such as (**Ei**sen)(b**a**hn)(kn**o**ten)(p**u**nkt)(h**i**nund)(h**e**r)(sch**ie**ber), for instance, is one phonological phrase that contains 11 syllables and 7 feet of which the stressed syllables are given in boldface. The prediction again is that simplex words can never contain more than 7 feet.

2 Evans (1995) shows that Kayardild for instance contrasts a laminodental [t̪], an apicoalveolar [t], a lamino-prepalatal [c] and a retroflex [ʈ] voiceless coronal plosive.

3 It should be observed that [v] (Butskhrikidze, 2002, p. 109) is considered to be a secondary articulation on the preceding consonant, so that the longest surface sequence contains maximally five consonants. For a more detailed account we refer to Butskhrikidze (2002) where it is argued that these surface sequences can arise by the deletion of a vowel or by adding a consonantal affix.

4 The term surroundings is used to avoid a confusion with the physical environment. Surroundings contains more than the environment. We will come back to that in section “Focus versus Memory” and in the section “Discussion.”

5 The term Focus is used to avoid confusion with other terms in cognition. We would like to emphasize here that Focus is not Attention. We will come back to that in the section “Discussion.”

6 www.brepolis.net

7 Although we did not found it in Latin, it is understandable that a rhythm in the pronunciation can avoid dissimilation by breaking the stem into parts or disjoining the stem and the suffix.

8 Surely k can change into over a long period, but only if k changes into g and the status of this consonant changes from a –C representation into a +V representation. This is a different and historical process (cf. Jacobs, 2019).

9 Notice, that (re)structuring the content of the brain may lead to what we call creativity in common parlance.

10 C2C5 and C3C4 are complementary representations as well, but with regard to the point to be made here, that does not alter the argumentation.

11 Notice that C2 and C3 refer to complex structures, if a subject would try to retain numbers heard before.

12 A maximum of span 7 is a smart choice of biology. A span of 8 or more would explode the possible solutions and sometimes require 3 instead of 2 complementary representations at the same time. It could lead to uncertainty and overload.
